# Pushing at the Boundaries of Pterin Chemistry

**DOI:** 10.3390/molecules29194587

**Published:** 2024-09-27

**Authors:** Jevy V. Correia, Siva S. M. Bandaru, Carola Schulzke

**Affiliations:** Bioanorganische Chemie, Institut für Biochemie, Universität Greifswald, Felix-Hausdorff-Str. 4, 17489 Greifswald, Germany; jevy.correia@uni-greifswald.de (J.V.C.); siva.bandaru@uni-greifswald.de (S.S.M.B.)

**Keywords:** pterin, tosylation, Sonogashira cross-coupling, Suzuki cross-coupling, solid-state mechano-synthesis

## Abstract

Pterins are molecules of substantial interest as they occur in nature in a number of forms with quite distinct and often indispensable roles. Chemically, the synthesis of the principle pterin scaffold is comparably simple, while the insolubility of the pterin building block renders synthetic derivatization extremely difficult. When aiming at modeling naturally occurring pterins of extended chemical structure, this is a considerable problem. A notable set of strategies was developed in the course of the present study, which are able to overcome the lack of reactivity of the pterin backbone. These include a strategic choice regarding protection groups, uncommon chemical transformation, ball milling and combinations thereof. Some novel pterins with quite distinct substitution motifs were successfully synthesized and characterized by spectroscopic and spectrometric analyses as well as single-crystal structural analyses for three of them.

## 1. Introduction

**Figure 1:**
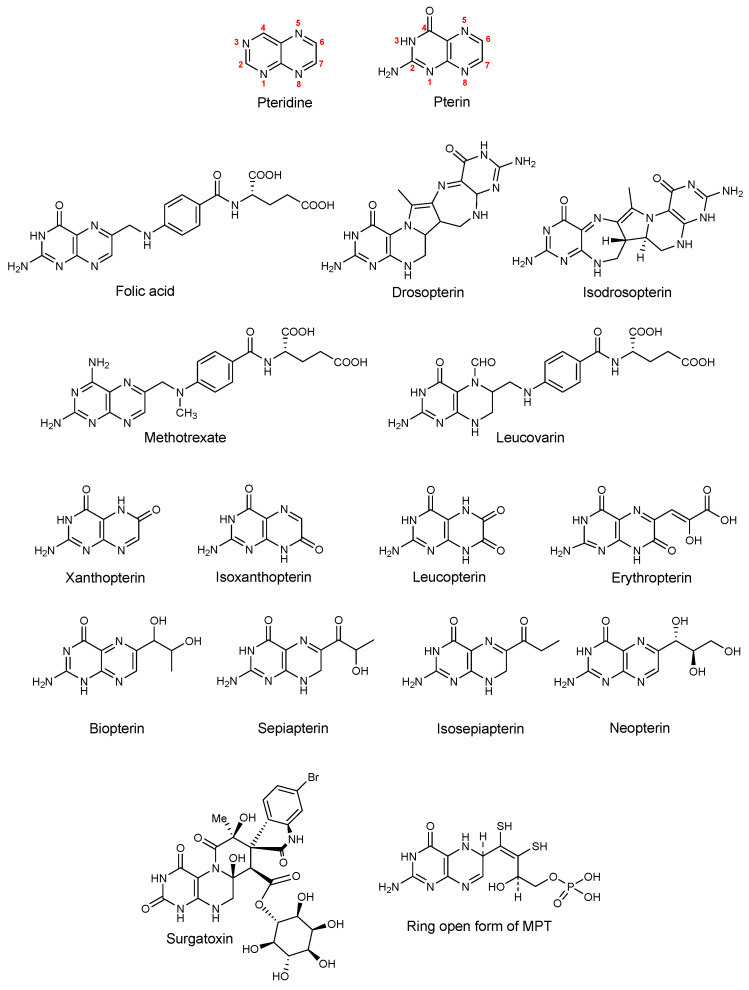
Molecular structures of examples of biologically important pterin derivatives.

The pterin structural motif is ubiquitous in nature and comprises a critically important moiety in various biologically active molecules (Figure 1). The name ‘pterin’ is derived from the word “pteron”, which is Greek for wings, accounting to their presence as pigments in wings of butterflies [1]. Pterins belong to the family of pteridines, which are low-molecular-weight heterocyclic compounds consisting of a pyrimidine ring fused to a pyrazine ring (Figure 1). Pterins, among the pteridine heterocycles, are defined by an amino group at position 2 and a keto group at position 4. They form the most common class of naturally occurring pteridines.

The documented chemistry of natural pterins began in 1889 when Hopkins isolated yellow purine-like pigments from the wings of the common English brimstone butterfly [2]. These pigments, after being re-isolated in pure form by Schöpf and Wieland in 1925–26, were named xanthopterin [3] and leucopterin [4], respectively. Subsequently, isoxanthopterin and erythropterin [5] were isolated from tropical butterflies. The structures of these pigments remained unknown for a long time due to their unusual physical properties and the difficulty in obtaining accurate elemental analyses due to incomplete combustion. The structure elucidation of xanthopterin, isoxanthopterin and leucopterin in 1940 through logical synthesis by Purrman [6] revived research in the fields of isolation and chemical synthesis of (new) naturally occurring pterins.

Pterins are integral components of life as they fulfill diverse biological roles. Key examples of pterins in biological systems include one-carbon transfer cofactors (such as folic acid and tetrahydrofolate) [7], redox cofactors (including biopterin [8,9,10,11] and neopterin [12]) and mollusk-derived toxins [13]. One representative example for the latter is surgatoxin, a structurally complex pterin isolated from the Japanese Ivory Mollusks found in the Surga Bay (Figure 1) [14]. Folic acid (Figure 1), one of the most essential human nutrients, possesses a pterin backbone and plays a significant role in the biosynthesis of amino acids and nucleic acids [7]. When activated to its tetrahydro form in vivo, the folate molecule can carry an activated methyl group at N5, which can be transferred to the substrate and that is essential for DNA synthesis. This is specifically important for swiftly dividing cells such as hair follicles, red blood cells, bone marrow cells, intestinal mucosa, and also for cancer cell proliferation, since these cells are all rapidly replicating their DNA. In consequence, methotrexate, as a pteridine derivative, is capable of inhibiting the activation of folic acid to folate by specifically binding to the enzyme dihydrofolate reductase (DHFR), which thereby acts as a quite powerful anti-cancer drug [15]. Leucovorin is another pterin-based drug molecule often used together with methotrexate in order to reduce the side effects of a respective treatment through a phenomenon which has been termed “leucovorin rescue” [16].

Neopterin (Figure 1), a redox co-factor, is often used as a marker in cancer patients, as its levels are found to be elevated due to incipient immune response [17,18]. It induces apoptosis and protects cellular proteins, rendering it one quite important molecule in humans.

Due to their heteroaromatic structures, many pteridines exhibit fluorescence, facilitating their detection even at low concentrations [19,20]. Besides widely distributed tetrapyrroles, pterins are among the most significant natural monomeric chromophores in various animals and insects. They contribute to the pigmentation of insect wings, the eyes and skin of fish and reptiles, and are even found in human urine. The eye coloration of *Drosophila melanogaster* (fruit fly) is due to multiple pterin molecules, including a dimeric pterin named drosopterin (Figure 1) [21]. Drosopterin and its isomeric forms, such as isodrosopterin and neodrosopterin, exhibit orange to red hues [22]. Other pterins, like yellow sepiapterin and isosepiapterin, are also present in *Drosophila* [23].

Considering all this, pterins are consequently of substantial medicinal significance. They have, therefore, become a notable topic of investigation in life sciences, medicine, pharmacology, the pharmaceutical industry and, more generally, the fields of biochemistry and chemistry. With regard to the latter, the context of synthesizing mimics of the molybdenum cofactor (Moco) and molybdopterin (MPT) is noteworthy and constitutes a specific core research area of our group.

The molybdenum co-factor (Moco) is an inherent part of almost all molybdenum-dependent enzymes (the exception being nitrogenase enzymes), and these oxidoreductases significantly drive the global C, N, S and As cycles [24]. Deficiencies of Moco in humans go back to genetic disorders, which typically lead to early infant death caused by the accumulation of redox active sulfite due to the absence of sulfite oxidase (SO), among other biological/medicinal effects [25]. In the absence of SO, sulfite cannot be oxidized to sulfate, which is the only path towards excretion for all sulfur atoms that enter the human metabolism and catabolism.

The rather uncommon chemical characteristics (which are notably distinct from, on paper, closely related heterocycles) and the pronounced low solubility of pterins pose challenges in the chemical transformations required for derivatizing chemically accessible precursors and synthesizing important target pterin scaffolds. Further, the reaction conditions and the transformations of pterins are very much substrate-specific, and a subtle change in one functional group substituent more often than not leads to a partial or, in some cases, even complete reversal of the chemical reactivity in certain positions.

Several examples of pterins substituted at positions 6 and 7 (see Figure 1 for numbering) are known in the literature, and 7-substituted pterin derivatives are fairly easy to access due to the regio-specific preference towards the formation of the 7-isomer owing to the electronic distribution in the pterin molecules. The Minisci acylation is an example where position 7 is regio-specifically preferred over position 6, leading to the dominant formation of 7-acylpterins [26]. Though derivatization of position 7 is more feasible, most of the naturally occurring, biologically important pterin derivatives are found to be substituted at position 6. It is, therefore, a necessity to design and develop synthetic methodologies allowing easier access to 6-substituted pterin derivatives.

Traditionally, throughout the literature, 6-substituted pterin derivatives have been synthesized either using a specific aliphatic molecule under acidic conditions for condensation with the pyrimidine precursor (Gabriel–Isay or Polovonoski–Boon synthesis) [27,28,29] or by using a pyrazine ring already bearing a substituent at position 6 for the synthesis via the Taylor method [30,31]. These approaches significantly constrain the selection of substituents that can be introduced, thereby limiting the accessibility of novel 6-substituted pterin derivatives.

Another synthetic route for the preparation of 6-substituted pterins is through the synthesis of 6-chloropterin derivatives, followed by further derivatization through cross-coupling reactions (or other related chemical transformations). The respective synthetic approach was first reported by Wolfgang Pfleiderer; it proceeds through the synthesis of 2-aminopteridin-4-one, followed by the synthesis of *N*-oxide and chlorination of the molecule at position 6 using acetyl chloride and trifluoracetic acid at −40 °C [32]. This traditional route used for the synthesis of 6-chloro derivatives of pterins is considerably long (requiring multiple steps) and inefficient in terms of yields. The atom economy of the overall process is, hence, sub-optimal to put it mildly.

As an alternative to this strategy, we proposed the synthesis and derivatization of the 6-tosyl derivative of a pterin (compound **2**) synthesized through 6-oxopterin (compound **1**)— or possibly from xanthopterin in future endeavors. This approach was inspired by related experiments by Winston Nxumalo and Andrew Dinsmore [33] on pteridine heterocycles. Similarly, a sulfonyl pterin was reported by the group of Shizuaki Murata that could be successfully prepared from a 6-triflate pterin derivative [34]. However, the synthetic route applied, notably, merely follows the traditional multistep procedure already reported as used for the synthesis of 6-chloropterin derivatives.

Compound **2** was subsequently successfully investigated for applications in Sonogashira and Suzuki cross-coupling reactions, which facilitates comfortable access to a considerably larger family of respective pterin derivatives which may be exploited in the biological and/or medicinal context in the future.

In addition, further important transformations targeting modifications of interest based on the natural template molecules were addressed. For instance, the synthesis of the pyranopterin heterocycle through condensation of a pyran bromohydrin with a pyrimidine derivative was attempted to yield a tetrahydro form of pyranopterin (as a better model for naturally occurring molybdopterin). The synthesized tetrahydro form was found to be unstable, and the accessibility and potential stabilization were further investigated via reduction reactions on different pterin analogues.

The here-reported work opens doors towards more various, more facile and more efficient derivatization of pterins in general but also emphasizes once again that the respective chemistry is difficult and that the reduced species must be expected to be fragile in nature, all of which is described and discussed in this contribution.

## 2. Results and Discussion

### 2.1. Cross-Coupling Reactions of 6-Pseudohalopterin Derivatives with Different Substrates

Our interest in pterin chemistry stems from the aim of modeling the prosthetic group of molybdenum- and tungsten-dependent enzymes as closely as possible in order to generate respective artificial active sites. The various attempted syntheses in this context turned out to be considerably more challenging than originally anticipated, and progress has been merely incremental over the years. One particular requirement for the purpose was to obtain the target compounds with a free amino function attached to position C-2, as this is part of an important key–lock interaction with the protein scaffold of the active site environment. Most often, pterin derivatives which are published have a protection group on this amine that would render syntheses more complicated (additional steps for protection and de-protection) or generally not applicable for our cause. Working without any protection, however, magnifies the notorious solubility problem often detrimentally associated with pterin chemistry. As a common feature of our molecules, a pentyl substituent is present at the oxygen function attached to position C4, which locks the tautomerism to the side of the imine-ol component (rather than the amine-one species). This increases the solubility of the intermediate compounds often, yet not always, sufficiently enough for further transformations and does not interfere with the key functions regarding cofactor characteristics and important intermolecular interactions with the protein. A specific position on the pterin scaffold which we need to be able to variably functionalize is position C-6. Ideally, we could simply couple an ene-dithiolate moiety to this carbon and have our target ligand molecule; based on numerous respective experiments, this appears to be not possible though, which leads us to explore further options for derivatization in this position to eventually reach our targets, albeit by a detour approach. A milestone in this regard would certainly be the facile and economic access to a C-6 halo- or pseudo-halo derivative. This was indeed successfully realized in the course of this work.

#### 2.1.1. Synthesis of 6-Tosyl Pterin

**Figure 2:**
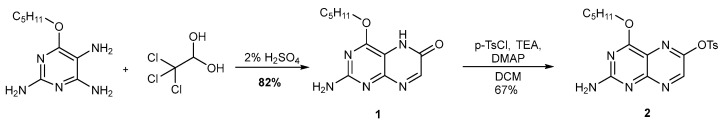
Reaction scheme for the direct synthesis of the 6-oxo-pterin derivative compound **1** and subsequently of the tosylated derivative thereof (compound **2**) based on the adaptation of literature procedures [35,36]. Instead of multiple steps towards a pterin with an activated C-6 position, only two steps are required herewith.

One strategy towards accessing the targeted 6-chloropterin or 6-tosylpterin derivatives in an atom-efficient way would be through the initial synthesis of 6-oxopterin as an intermediate species. Various different literature-reported procedures applied to pteridine and also some pterin derivatives using glyoxalic acid precursors for condensation with the diamino-pyrimidine starting material proved either unsuccessful or excessively inefficient with regard to yields, purification requirements and results. This problem was eventually solved for pteridines by the work of Bergmann et al., who synthesized what are essentially more-or-less distant relatives of compound **1** successfully via the condensation of 2,4,5-triamonopyrimidine with chloral hydrate (see Figure 2 for this strategy being applied to our own specific precursor derivative) [35].

Compound **1** was prepared according to this synthetic approach and then subsequently tosylated by the adaptation of literature-known reaction conditions to yield the most important and central molecule of this study, compound **2**, in a 67% yield (Figure 2) [36]. The overall reaction times are substantially decreased in comparison to previously reported methods (achievable within a time frame of two days, with one overnight reaction for the second transformation required, compared to the known procedures with multiple steps, some of which alone requiring a number of days of reaction time). Most notably, compound **2** can be isolated simply via crystallization out of the dichloromethane solvent by the addition of hexane and is thereby obtainable directly in considerable analytical purity (no column chromatography required). In consequence, the synthetic and isolation methodology applied allows fast, facile and atom-economic access to compound **2**, which can be further used for applications in different chemical transformations including but not limited to Sonogashira cross-coupling and Suzuki cross-coupling reactions, as described below. This is in stark contrast, for instance, with regard to previously published protocols which were aimed at chlorination of the 6-position and has thereby the obvious potential to be an actual game-changer procedure for pterin derivatization applications.

**Figure 3:**
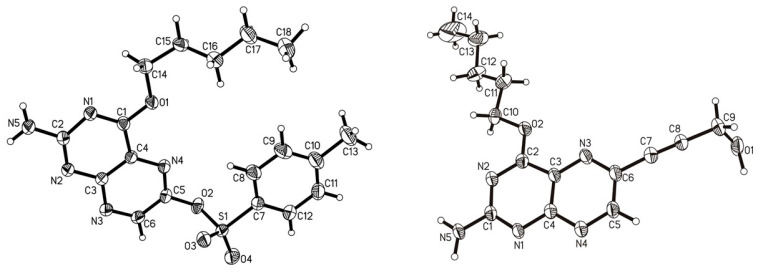
Molecular structures of compounds **2** and **3**. Only one disordered component of each structure is shown. Ellipsoids are at the 50% probability level.

Compound **2** crystalized into considerably large single crystals through the slow evaporation of the solvent from a solution in DCM, and single-crystal X-ray analysis revealed a triclinic cell setting with a *P*1; ¯ space group (Figure 3). The pentyl substituent on the pterin oxygen in position C-4 (imine-ol tautomer) is disordered severely, which could be modeled quite sufficiently but also led to the presence of two distinct (still disordered) molecules in the asymmetric unit. Likely, the considerable flexibility of this chain, as even apparent in the solid state, is a defining factor with regard to the most often sufficient solubility of related species. The entire rest of the molecule is unaffected by crystallographic problems, the overall data quality is very good, and the chemical structure, hence, unambiguous.

#### 2.1.2. Sonogashira Cross-Coupling Reactions Employing Compound **2**

There are previous reports available with regard to Sonogashira cross-coupling reactions of 6-chloropterin and -pteridine analogues, and the synthesized alkynyl pterin and pteridine derivatives have indeed been applied in hydration reactions to access naturally occurring pterin molecules such as sepiapterin or for the synthesis of pterin-bearing dithiolene ligands [37] or Moco mimics [38]. However, considering the specific research goals of our group, the amino function on C-2 of the pterin should be free (see above) in the eventual target molecules, and, in order to avoid resource costly protection and de-protection procedures, we attempted this synthetic approach foregoing the said protection strategies. With our new precursor/intermediate compound **1**, the following transformations do indeed tolerate this free amino group, and the entire series of processes is considerably more convenient than what has been described in the context of related chemistry before.

**Figure 4:**
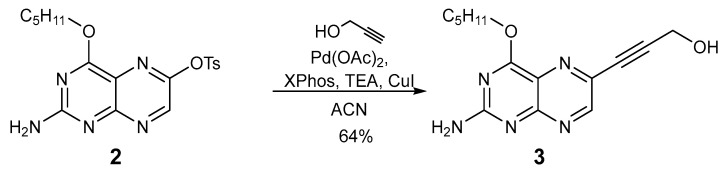
Reaction scheme for the synthesis of the 6-proporagyl-pterin derivative compound **3** based on the adaptation of a literature procedure [33].

The Sonogashira cross-coupling reactions of compound **2** with propargyl alcohol were optimized starting from literature-reported protocols [33] by applying various specific combinations of conditions. The reaction of compound **2** with propargyl alcohol in the presence of 5% Pd(OAc)_2_, XPhos, 5% CuI and trimethylamine resulted eventually in the formation of the cross-coupled product compound **3** in satisfying yields (64%; Figure 4). Compound **3** was characterized by NMR spectroscopy and APCI mass spectrometry. Also, single crystals of compound **3** could be obtained from the slow evaporation of a solution in dichloromethane. Single-crystal X-ray structural analysis of the grown crystals again evidenced a triclinic cell setting with a *P*1; ¯ space group (see Figure 3 above for the molecular structure), and, as in compound **2**, the pentyl side-chain is disordered (albeit only in the three terminal carbon centers), while the rest of the molecule is very well resolved.

#### 2.1.3. Suzuki Cross-Coupling Reactions of Compound **2**

Pterins exhibit notable redox properties, and several pterin-based drug molecules are currently employed as anticancer agents (see above). Additionally, numerous pterin-derived compounds are undergoing clinical trials for various pharmaceutical applications. The Suzuki cross-coupling of pterin derivatives generally provides an alternative route towards the synthesis of such pharmaceutically important pterin derivatives given that suitable precursor pterins are available. In fact, 6-aryl pterin derivatives synthesized via the Suzuki cross-coupling of 6-chloropterin precursors have been investigated for their immuno-suppressive [39], anti-inflammatory [40] and antiviral activities [41].

In order to investigate whether respective reactivity could also be realized with the tosylated pterin derivative compound **2**, literature-known reaction conditions were consequently tested with this new and easily accessible pterin precursor. The 6-tosyl pterin compound **2** was reacted with 4-methoxyphenylboronic acid under Suzuki cross-coupling conditions. Various combinations of Pd(OAc)_2_ and Pd(COD)_2_Cl_2_ catalysts, dppf and SPhos ligands and DMF and water as solvents were tested at temperatures ranging from room temperature to 120 °C. However, the desired cross-coupled product could not be obtained in any of these various attempts, while the major side product identifiable was the de-pseudohalogenation product.

Considering these many failed attempts and the notoriously limited solubility of pterins in organic solvents, an entirely different procedure was subsequently applied. The notable work by Kubota and Hajime and co-workers served as inspiration, who applied solid-state mechano-chemical strategies to Suzuki reactions [42].

**Figure 5:**
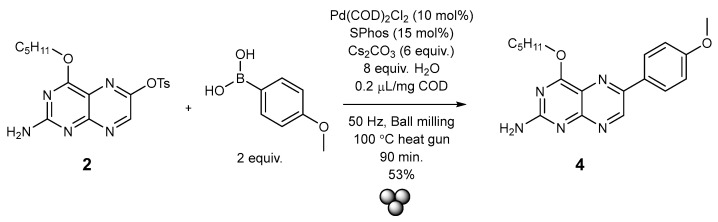
Reaction scheme for the Suzuki cross-coupling of compound **2** to the arylated pterin derivative compound **4**.

**Figure 6:**
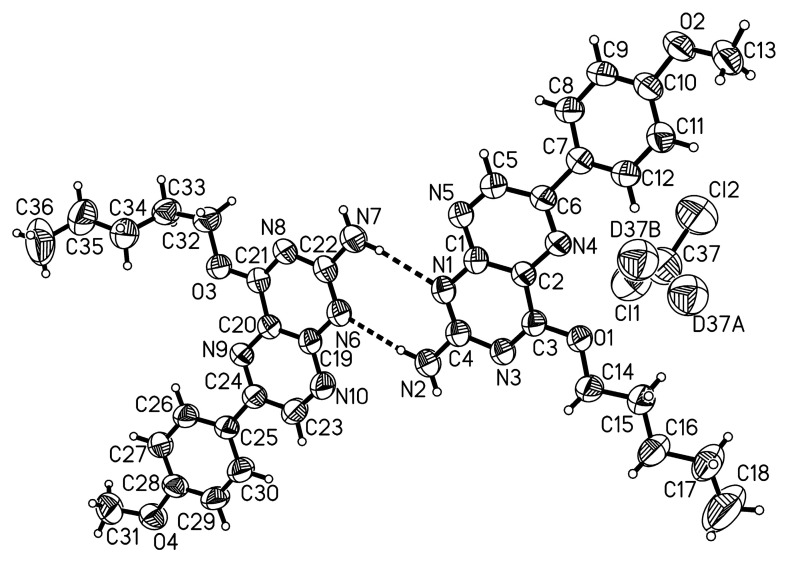
Molecular structure of the arylated pterin derivative compound **4**. Only the major components of the disorders are shown. Ellipsoids are presented at the 50% probability level.

We therefore also employed the mechano-chemical methodology for the Suzuki cross-coupling of the 6-tosyl pterin **2** together with Pd(COD)_2_Cl_2_, SPhos and Cs_2_CO_3_. All these solid reactants/catalysts were loaded into a stainless steel jar along with the 6-tosyl pterin and 4-methoxyphenylboronic acid under ambient conditions (Figure 5). Water and 0.2 µL/mg of COD were added as additives, and two 9 mm stainless steel balls were introduced to the jar. The reaction vessel was sealed with a polyolefinic waxed film (Parafilm©) and milled at 50 Hz frequency at 100 °C using a heat gun pointed at the vessel. After 90 min, the mixture was left standing to cool to room temperature and then subjected to chromatographic purification. The cross-coupled product was isolated with a 53% yield and characterized by NMR spectroscopy and Mass spectrometric analysis. Eventually, crystals had formed in the NMR tube, and an X-ray structural analysis could confirm the chemical structure of the product unambiguously (Figure 6). The compound also crystallized in triclinic space group *P*1; ¯ with two molecules plus one dichloromethane-d2 in the asymmetric unit. The two molecules are engaged in a strong bi-directional hydrogen bonding interaction (see Figure 6). The data quality of this crystal suffers to some extent from the measurement at r.t. but it comprises still undisputable proof that the reaction was successful. Again, the pentyl protection group chains are disordered, as is the co-crystallized solvent, while the residual molecule parts are all well behaved.

It was, hence, possible to employ the tosylated pterin compound **2** quite economically for further functionalization in position C-6 via Suzuki cross-coupling using mechano-chemistry as a new strategy in pterin functionalization. This methodology may be applied to various other boronic acid derivatives in the future and thereby opens the door wide to the facile access to a whole respective distinctly functionalized pterin family.

### 2.2. Accessing Tetrahydropyranopterin through Condensation Reactions

**Figure 7:**
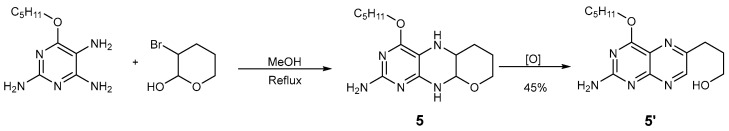
Reaction scheme for the synthesis of the transient tetrahydropyrano pterin derivative **5** and its spontaneous oxidation to the open-ring form compound **5′**.

In the active sites of the molybdenum and tungsten dependent enzymes, the molybdopterin ligand has been found in distinct forms, with an oxidized central pyrazin ring (as in most of our pterins), as di-hydro and as tetrahydro species [43], while the latter is the most commonly found component. Condensed to this central N-heterocycle is an ene-pyran in molybdopterin. The synthesis of pyranopterin, as a more accurate model of the natural molybdopterin ligand of the molybdenum cofactor, was attempted through the condensation reaction of 6-(pentyloxy)pyrimidine-2,4,5-triamine with 3-bromotetrahydro-2H-pyran-2-ol, and the formation of 6,7-tetrahydropterin derivative **5** was indeed observed (Figure 7). 3-bromotetrahydro-2H-pyran-2-ol was synthesized employing a literature-reported protocol [44], and the condensation reaction with 6-(pentyloxy)pyrimidine-2,4,5-triamine was carried out by refluxing in methanol for 2 h. The isolation of the tetrahydropyranopterin derivative **5** was, however, not successful. After several repetitions of the synthesis and respective attempts of isolation, it was eventually noticed that the targeted product is inherently unstable based on its facile oxidation. The reduced tetrahydro form of the pyranopterin was continuously and eventually completely oxidized, which was facilitated by the ring opening of the associated pyran ring as well as aromatization of the pyrazine ring. Albeit, the initial formation of the reduced tetrahydropyranopterin derivative was unambiguously confirmed by APCI mass spectrometry. The oxidation and aromatization of the terahydropyran derivative were further accelerated under oxidative conditions using selenium dioxide as the oxidizing agent to specifically obtain compound **5′** in a 45% yield and prove that it is indeed the decomposition product of **5**. NMR spectroscopy and APCI mass spectrometric analysis of compound **5′**, hence, indirectly confirmed the formation and transient nature of compound **5**. Distinct purification procedures under various conditions as well as further in situ chemical transformations are currently being explored in order to eventually isolate a respective reduced pterin-pyran derivative, as of yet without success, unfortunately.

### 2.3. Reduction of the Pyrazine Ring of Pterins

Tetrahydropterin (or reduced pterin) derivatives are further known as biologically active molecules which have important metabolic roles as redox and one-carbon transfer cofactors. Many drug molecules are known that possess a tetrahydropterin backbone. Tetrahydrobiopterin, for instance, is an essential co-factor for some aromatic monooxygenases responsible for the synthesis of tyrosine, L-Dopa and 5-hydroxytryptophan [45,46,47]. A lack of respective co-factors causes several neurological disorders, including Parkinson’s disease and atypical phenylketonuria [48,49]. Consequently, pterin derivatives in their tetrahydro form are of general interest for potential applications for the successful treatment of these conditions.

**Figure 8:**
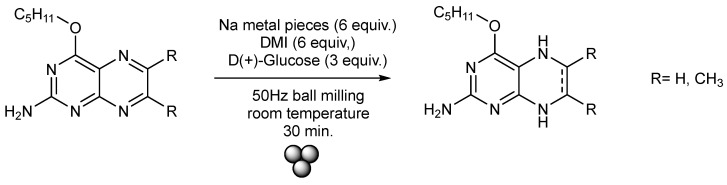
Reaction scheme for the mechano-chemical synthesis of the unstable di-hhydro, tetrahydropyrano pterin derivatives which, as of yet, could not be properly purified.

Since the direct synthesis of a reduced form bearing the pyran structural motif had turned out to not be feasible for its inherent instability towards oxidation, it appeared reasonable to try doing the opposite, i.e., access the tetrahydro form via reduction of an oxidized species lacking the pyran moiety. By this, it should be possible to access pharmaceutically important molecules in a more controllable manner. Though some procedures for the reduction of pterin molecules have been reported, most of these methods use hydrogen gas and expensive metals like platinum and palladium as reducing agents and additionally require very high temperatures and the use of an autoclave [50,51] which is not always readily accessible. Considering the success with the solid-state Suzuki cross-coupling (see Section 2.1.3), in this work we have also tested the more economic reduction of pterin heterocycles using elemental sodium metal and glucose as reducing agents under mechano-chemical conditions at room temperature (Figure 8).

Inspired by a recent report about the mechano-chemical Birch reduction of aromatics, we investigated similar reaction conditions for the reduction of pterin precursors with comparably easy-to-handle reducing agents [52]. Adopting the reported conditions, we examined both 6,7-unsubstituted and 6,7-dimethyl-substituted pterin substrates. The respective pterin precursor was combined with small pieces of sodium metal, D(+)-glucose and DMI in a metal jar. The mixture was sealed and ball-milled with an 8 mm stainless steel ball at room temperature for 30 min. Post-reaction analysis using APCI mass spectrometry of the resulting reaction mixtures confirmed the formation of 5,6,7,8-tetrahydropterin in the case of the unsubstituted pterin and 5,8-dihydropterin in the case of the 6,7-dimethyl-substituted pterin (see SI for spectroscopic evidence).

Not entirely unexpectedly, the targeted products, however, decomposed rapidly into unknown substances during the purification processes. We are currently working on (i) optimizing the purification procedures (searching for product handling which completely avoids exposure to air/oxygen after preparation) and (ii) a better understanding of the underlying causes of the compounds’ instabilities so that it will be possible in the future to stabilize these essential reduced pterin species by variations of their electronic structures (e.g., by employing distinct substitution motifs).

## 3. Materials and Methods

### 3.1. Materials, Methods and Instrumentation

#### 3.1.1. General Experimental Procedures

The majority of experiments, unless otherwise stated, were carried out either under nitrogen or argon atmosphere using standard Schlenk techniques. Reagents and starting materials were used as purchased without further purification. Dimethylformamide (DMF) was dried by refluxing under argon for 48 h over P_2_O_5_. Purification through column chromatography was conducted using silica gel VWR (particle size of 0.063–0.200 mm, 70–230 mesh ASTM). 6-(Pentyloxy)pyrimidine-2,4,5-triamine [32] and 3-bromotetrahydro-2H-pyran-2-ol [44] were synthesized using literature-reported procedures. The substrates for reduction of pterins, 4-(pentyloxy)pteridin-2-amine [53] and 6,7-dimethyl-4-(pentyloxy)pteridin-2-amine [54], were similarly synthesized based on reported literature. ^1^H- and ^13^C-NMR spectra were recorded on a Bruker Avance II 300 MHz spectrometer (300, 75.5 MHz, respectively) or on a Bruker Avance III instrument at 400 MHz (^1^H) and 101 MHz (^13^C), respectively, using CDCl_3_ dried over activated zeolites or DMSO-d6 as solvent (Bruker Biospin GmbH, Rheinstetten, Germany). Chemical shifts (δ) are given in parts per million (ppm). ^1^H-NMR spectra were referenced to the peaks of residual protons of the deuterated solvent; ^13^C-NMR spectra were referenced to the deuterated solvent itself. Multiplicities are abbreviated as follows: s, singlet; d, doublet; t, triplet; q, quartet; m, multiplet; *J*, coupling constant (Hertz). For assignments based on the chemical structures see the Supplementary Materials. Elemental analyses (C, H, N, S) were carried out with an Elementar Vario MICRO Cube elemental analyzer. Mass spectra were acquired with an Advion Expression CMS.

#### 3.1.2. Single-Crystal X-ray Diffraction

The data of single crystals of compounds **2** and **3** were collected at 170 K on a STOEIPDS 2T diffractometer with graphite-monochromated Mo-Kα-radiation (λ = 0.71073 Å). The samples were mounted on a glass fiber. Single-crystal X-ray diffraction data of compound **4** were recorded at room temperature on an XtaLAB Synergy diffractometer from Rigaku, with mirror monochromated Cu-Kα-radiation (λ = 1.54184 Å) and a hybrid pixel array detector (HyPix). Samples were mounted on LithoLoops produced by Molecular Dimensions fixed on pins produced by Hampton Research. Absorption corrections were performed using X-Red32 and X-Shape (by STOE & Cie GmbH 2010, Darmstadt, Germany) in the case of the Mo source or CrysAlisPro (Rigaku OD, 2022, Tokyo, Japan) in the case of the Cu source. All structures were solved by dual methods (SHELXT-20118) and refined by full-matrix least-squares techniques using the SHELXL executable and the WingX GUI [55,56,57]. All non-hydrogen atoms were refined with anisotropic displacement parameters. The carbon-bound hydrogen atoms, unless stated otherwise, were refined isotropically at calculated positions using a riding model, with their *U*_iso_ values constrained to 1.2 times *U*_eq_ of their pivot atoms for aromatic or methylene hydrogen atoms and to 1.5 times *U*_eq_ for the methyl atoms. For the cif-file of compound **2**, there are some B-alerts regarding the cell symmetry. These do not appear while the disordered pentyl substituents are refined as only a single component (without disorder modeling), i.e., the relatively low symmetry and the presence of two molecules in the asymmetric unit go back to differences in the pentyl moieties. The disorders were modeled with SAME, SIMU and DELU constraints (at default values). The two hydrogen atoms on each of the molecules’ amine function and the single hydrogen on the pterins were refined freely. In the case of compound **3**, the N and the O bound hydrogen atoms were refined freely. The data for compound **4** were collected at room temperature because the cooling system was and is not operational. The data quality is, hence, not great. There are two molecules of compound **4** refined in the asymmetric unit together with one dichloromethane-d2 solvent. The pentyl side chains are disordered in both molecules. This was modeled with SADI, SAME, SIMU and DELU constraints. The disordered solvent was modeled with DFIX, SAME, SIMU and DELU constraints. One reflex was omitted as a clear outlier. The amine hydrogen atoms were located and refined almost freely, with only the N-H distances being constrained with SADI.

Crystallographic data were deposited with the Cambridge Crystallographic Data Centre, CCDC, 12 Union Road, Cambridge CB21EZ, UK. These data can be obtained free of charge on quoting the depository numbers CCDC 2375007 (**2**), 2375008 (**3**) and 2375325 (**4**) by email (deposit@ccdc.cam.ac.uk) or via their web interface (at http://www.ccdc.cam.ac.uk).

### 3.2. Syntheses

*Synthesis of 2-amino-4-(pentyloxy)pteridin-6(5H)-one, compound* **1**: 6-(Pentyloxy)pyrimidine-2,4,5-triamine (2.41 g, 11.4 mmol) was dissolved in 2% H_2_SO_4_ and the solution stirred at 100 °C for 15 min. A solution of chloral hydrate (3.77 g, 22.8 mmol, 2 equiv.) in 10 mL water was added to the pyrimidine solution and the reaction mixture stirred at 100 °C for an additional 15 min. The reaction mixture was allowed to cool to room temperature. The precipitate which had formed was filtered under vacuum, washed with water and dried under air to give compound **2** as an orange solid (yield: 2.346 g, 82.5%). ^1^H NMR (DMSO-d6, 300 MHz): δ = 8.50 (s, 1H, 7-CH), 4.54 (t, *J* = 6.6 Hz, 2H, 11-CH_2_), 1.79 (q, *J* = 1.0 Hz, 2H, 12-CH_2_), 1.25–1.45 (m, 4H, 13,14-CH_2_), 0.90 ppm (br t, *J* = 7.0 Hz, 3H, 15-CH_3_). ^13^C NMR (DMSO-d6, 75 MHz): δ = 158.5, 157.1, 155.4, 143.6, 117.7, 95.0, 69.2, 27.6, 27.4, 21.8, 13.9 ppm. (+ve) APCI-MS m/z = 250.3 m/z calcd. for C_11_H_15_N_5_O_2_ [M + H^+^]; found: 250.4 [M + H^+^]. CHNS calcd for C_11_H_15_N_5_O_2_: C, 53.00; H, 6.07; N, 28.10; found: C, 52.60; H, 6.47; N, 27.70; Melting point: 225.7 °C.
*Synthesis of 2-amino-4-(pentyloxy)pteridin-6-yl 4-methylbenzenesulfonate, compound* **2**: 2-Amino-4-pentyloxypteridine-6-one, 1 (997 mg, 4 mmol), DMAP (48.95 mg, 0.4 mmol, 0.1 equiv.) and p-toluene sulfonyl chloride (762.6 mg, 29 mmol, 2 equiv.) were dissolved in DCM (15 mL) and cooled to 0 °C. Et_3_N (1.1 mL, 8 mmol, 2 equiv.) was added drop-wise to the mixture, and the resulting solution was allowed to warm to room temperature and stirred overnight. The reaction was quenched with sat. NaHCO_3_ (15 mL), the organic layer separated, and the aqueous layer washed with DCM (2 × 20 mL). The combined organic layers were dried over Na_2_SO_4_, and the solvent evaporated to dryness to give a reddish orange solid, which was recrystallized from DCM to give compound **2** as an orange powder (yield: 1.081 g, 67%). ^1^H NMR (CDCl_3_, 400 MHz): δ = 8.64 (s, 1H, 7-CH), 8.03 (d, *J* = 1.0 Hz, 2H, 2′,6′-ArCH), 7.36 (d, *J* = 8.0 Hz, 2H, 3′,5′-ArCH), 5.69 (br s, 2H, NH_2_), 4.50 (t, *J* = 6.9 Hz, 2H, 11-CH_2_), 2.47 (s, 3H, 4′-CH_3_), 1.91 (quin, *J* = 7.1 Hz, 2H, 12-CH_2_), 1.40–1.53 (m, 4H, 13,14-CH_2_), 0.97 ppm (t, *J* = 1.0 Hz, 3H, 15-CH_3_). ^13^C NMR (CHLOROFORM-d, 101 MHz): δ = 167.5, 161.7, 156.1, 148.5, 146.0, 145.4, 133.4, 129.9, 129.6, 119.8, 68.7, 28.5, 28.2, 22.6, 22.0, 14.2 ppm. (+ve) APCI-MS m/z = 404.46 m/z calcd. for C_18_H_21_N_5_O_4_S [M + H^+^]; found: 404.5 [M + H^+^]. CHNS calcd for C_18_H_21_N_5_O_4_S: C, 53.59; H, 5.25; N, 17.36; S, 7.95; found: C, 53.79; H, 5.45; N, 17.56; S, 8.15. Melting point: 167.1 °C.
*Synthesis of 3-(2-Amino-4-(pentyloxy)pteridin-6-yl)prop-2-yn-1-ol, compound* **3**: Compound **2** (807 mg, 2 mmol), Pd(OAc)_2_ (22.45 mg, 0.1 mmol, 5 mol%), X-Phos (95.34 mg, 0.2 mmol, 10 mol%) and CuI (19.1 mg, 0.1 mmol, 5 mol%) were added to an oven-dried Schlenk tube, and the solids were evacuated. Dry DMF (3 mL) was added to the reaction flask and the contents stirred for 10 min. Triethylamine (0.54 mL, 4 mmol, 2 equiv.) was added to the reaction mixture followed by drop-wise addition of propargyl alcohol (0.14 mL, 2.4 mmol, 1.2 equiv.). The reaction mixture was allowed to stir overnight at 80 °C. The reaction mass was filtered through celite, the DMF evaporated under vacuum, and the product was purified by column chromatography on aluminum oxide (neutral) using 2% CH_3_OH/CHCl_3_ (brown crystalline solid; yield: 367.56 mg, 64%). ^1^H NMR (DMSO-d6, 400 MHz): δ = 8.79 (s, 1H, 7-CH), 7.32–7.63 (m, 2H, NH_2_), 5.51 (t, *J* = 6.0 Hz, 1H, 10-OH), 4.46 (t, *J* = 1.0 Hz, 2H, 11-CH_2_), 4.37 (d, *J* = 1.0 Hz, 2H, 10-CH_2_), 1.75–1.88 (m, 2H, 12-CH_2_), 1.29–1.46 (m, 4H, 13,14-CH_2_), 0.90 ppm (t, *J* = 1.0 Hz, 3H, 15-CH_3_); ^13^C NMR (DMSO-d6, 75 MHz): δ = 166.4, 161.9, 155.6, 152.8, 132.2, 122.8, 92.2, 81.3, 67.4, 49.4, 27.8, 27.6, 21.8, 13.9 ppm ^13^C-dept 135NMR (DMSO-d_6_, 75 MHz): δ = 152.8, 67.4, 49.4, 27.8, 27.6, 21.8, 13.9 ppm. (+ve) APCI-MS m/z = 288.33 m/z calcd. for C_14_H_17_N_5_O_2_ [M + H^+^]; found: 288.2 [M + H^+^]. CHNS calcd for C_14_H_17_N_5_O_2_: C, 58.52; H, 5.96; N, 24.38; found: C, 58.32; H, 5.76; N, 24.28.
*Synthesis of 6-(4-methoxyphenyl)-4-(pentyloxy)pteridin-2-amine, compound* **4**: To a metal jar, 6-tosyl pterin 1 (200 mg, 0.5 mmol), 4-methoxyphenylboronic acid (152 mg, 1 mmol), 10 mol% Pd(COD)₂Cl₂ (14.3 mg, 0.05 mmol), 15 mol% SPhos (31 mg, 0.075 mmol), Cs₂CO₃ (977 mg, 3 mmol), 72 µL of H₂O and 40 µL of 1,5-cyclooctadiene were added, along with two 9 mm stainless steel balls. The mixture was sealed in ambient air and ball-milled at 50 Hz for 90 min at 100 °C using a heat gun. The heat gun, producing a substantially hot stream of air, was fixed at a distance which allowed the temperature of the metal vessel to be maintained stable throughout the milling process. After the reaction was complete, the crude product was purified by preparative thin layer chromatography (TLC) on silica using a 2% MeOH/CHCl₃ mobile phase. N.B.: Likely, the employment of alumina column chromatography will result in more efficient purification and better yield. For a lack of resources, it was not possible to test this in the course of this study, while various attempts using silica columns returned unsatisfactory results. The desired product was eventually isolated as a pale-yellow solid in a 90 mg, 53% yield. ^1^H NMR (300 MHz, CD_2_Cl_2_): δ = 9.22 (s, 1 H, 7-CH), 8.04 (m, *J* = 8.71 Hz, 2 H, 2′,6′—ArCH), 7.05 (m, *J* = 8.71 Hz, 2 H, 3′,5′—ArCH), 5.52 (br. s., 2 H, NH_2_), 4.57 (t, *J* = 6.92 Hz, 2 H, 11-CH_2_), 3.88 (s, 3 H, OCH_3_), 1.89–2.01 (m, 2 H, 12-CH_2_), 1.41–1.54 (m, 4 H, 13-CH_2_, 14-CH_2_), 0.96 ppm (t, *J* = 6.92 Hz, 3 H, 15-CH_3_); ^13^C NMR (75 MHz, CD_2_Cl_2_) δ = 168.5 (s, 1 C, C2), 161.7 (s, 1 C, C4), 161.6 (s, 1 C, C4′), 156.3 (s, 1 C, C6), 148.8 (s, 1 C, C9, C10), 128.7 (s, 1 C, C2′, C6′), 123.8 (s, 1 C, C1′), 115.0 (s, 1 C, C3′, C5′), 68.9 (s, 1 C, C11), 56.0 (s, 1 C, OCH_3_), 28.8 (s, 1 C), 28.6 (s, 1 C, C12, C13), 23.0 (s, 1 C, C14), 14.3 (s, 1 C, C15) ppm. APCI-MS (+ve) calculated—[M + H^+^] 340.4 m/z, experimental—[M + H^+^] 340.4 m/z. CHNS calcd for C_18_H_21_N_5_O_2_: C, 63.70; H, 6.24; N, 20.64; found: C, 63.95; H, 6.49; N, 20.89. Melting point: 189.6 °C (decomposed).
*Synthesis of 4-(Pentyloxy)-5a,6,7,8,9a,10-hexahydro-5H-pyrano [3,2-g]pteridin-2-amine, compound* **5**: A round-bottom flask equipped with a reflux condenser was charged with 2,5,6-triamino-4-pentyloxy pyrimidine and 3-bromotetrahydro-2H-pyran-2-ol. Methanol was added, and the reaction mixture was refluxed for 2 h. The progress of the reaction was monitored by TLC. Methanol was evaporated under vacuum and the yellow-colored solid residue used in the next reactions without further purification. (+ve) APCI-MS m/z = 294.38 m/z calcd. for C_14_H_23_N_5_O_2_ [M + H^+^]; found: 294.2 [M + H^+^].
*Synthesis of 3-(2-Amino-4-(pentyloxy)pteridin-6-yl)propan-1-ol*, **5′**: A residue of the preparation of compound **5** (1 mmol) was suspended in 1,4-dioxane (4 mL), and selenium dioxide (133.15 mg, 1.2 mmol, 1.2 equiv.) was added. The temperature was raised to 80 °C and the reaction mixture stirred overnight. The progress of the reaction was monitored by TLC and APCI mass spectrometry. 1,4-Dioxane was evaporated under vacuum and the product purified by silica gel column chromatography using 3% MeOH/CH_2_Cl_2_ as eluent (bright-yellow-colored solid; yield: 130 mg, 44.6%). ^1^H NMR (CDCl_3_, 300 MHz): δ = 8.68 (s, 1H, 7-CH), 5.57 (br s, 2H, NH_2_), 4.48 (t, *J* = 7.1 Hz, 2H, 11-CH_2_), 3.70 (t, *J* = 5.8 Hz, 2H, 10-CH_2_), 3.04 (t, *J* = 7.1 Hz, 2H, 8-CH_2_), 1.95–2.05 (m, 2H, 9-CH_2_), 1.85 (quin, *J* = 7.2 Hz, 2H, 12-CH_2_), 1.29–1.42 (m, 4H, 13,14-CH_2_), 1.16–1.22 (m, 1H, 10-OH), 0.87 ppm (t, *J* = 1.0 Hz, 3H, 15-CH_3_); ^13^C NMR (DMSO-d6, 75 MHz): δ = 166.6, 161.0, 155.6, 151.8, 151.2, 121.8, 67.1, 60.1, 32.2, 31.2, 27.8, 27.6, 21.8, 13.9 ppm. (+ve) APCI-MS m/z = 292.36 m/z calcd. for C_14_H_21_N_5_O_2_ [M + H^+^]; found: 292.6 [M + H^+^]. CHNS calcd for C_14_H_21_N_5_O_2_: C, 57.71; H, 7.27; N, 24.04; found: C, 58.11; H, 7.67; N, 24.44.

## 4. Conclusions

The synthesis of the tetrahydro form(s) of pyranoptein via a condensation reaction with a bromohydrin derivative provides an accessible route to pyranopterin, although further research into the synthesis and purification processes is ongoing, as the transient product’s instability still constitutes a problem, while the general chemical feasibility appears expandable. Further, the mechano-chemical reduction of pterin heterocycles using sodium metal and glucose represents a simple and cost-effective method for obtaining reduced pteridines, which are known to play significant biological roles. However, further investigations into purification procedures and stabilization strategies are required, which similarly constitutes ongoing work. The new pterin compound **2** being tosylated in the C-6 position was successfully synthesized in merely two steps and isolated with one, for pterin chemistry, uncommonly facile purification procedure, i.e., simple crystallization. This compound served (and will serve in the future) as an intermediate/precursor for the synthesis of novel pterin derivatives. That is, this precursor was successfully used as proof-of-principle in further chemical transformations, specifically, in Sonogashira and Suzuki cross-coupling reactions. The synthetic approach towards compound **2** utilizing the specific 6-oxo compound **1** as precursor all in all represents a novel and notably economic synthetic strategy. The procedure will facilitate the preparation of a wide range of pterin analogues based on the tosylate which is discussed in this work but possibly/likely also via triflates, which are currently under investigation by the group for Heck cross-coupling reactions. These synthetic methodologies applied to the preparation and subsequent transformations of compounds **1** and **2** provide an as-of-yet unexplored and very straightforward approach to various pterin derivatives with potential biological and pharmaceutical applications.

## Data Availability

The crystallographic data were deposited with the CCDC and are available by download via the deposition numbers (see experimental section). Spectra, if not part of the manuscript, are provided in the Supplementary Materials. Original data are available from the authors upon request.

## Supplementary Materials

The following supporting information can be downloaded at: https://www.mdpi.com/article/10.3390/molecules29194587/s1: NMR spectra of compounds **1**, **2**, **3**, **4** and **5′**; mass spectra of compounds **1**, **3**, **4**, **5′** and **5**; APCI mass spectra for reduction reactions; crystallographic data (crystal and refinement) for compounds **2**, **3** and **4**.
