# Supplementary material for: Pushing at the Boundaries of Pterin Chemistry
Source: Molecules. 2024 Sep 27;29(19):4587. doi: 10.3390/molecules29194587 (PMC11477544; doi:10.3390/molecules29194587)

Pushing on the boundaries of pterin chemistry

Jevy V. Correia[1], Siva S. M. Bandaru[1] and Carola Schulzke[1,*]

Supporting Information

[1] Universität Greifswald, Institut für Biochemie, Bioanorganische Chemie, Felix-Hausdorff-Str. 4, 17489 Greifswald, Germany; jevy.correia@uni-greifswald.de; siva.bandaru@uni-greifswald.de; carola.schulzke@uni-greifswald.de
* Correspondence: carola.schulzke@uni-greifswald.de; Tel.: +49 3834 420 43 21

Content:

NMR Spectra of compounds **1**, **2**, **3**, **4**, and **5'** Pages 2-8

Mass Spectra of compounds **1**, **3**, **4**, **5'**, and **5** Pages 9-10

APCI-Mass spectra for reduction reactions Pages 10-11

Crystallographic data (crystal and refinement) for compounds **2**, **3**, and **4** Pages 12-13

NMR Spectra

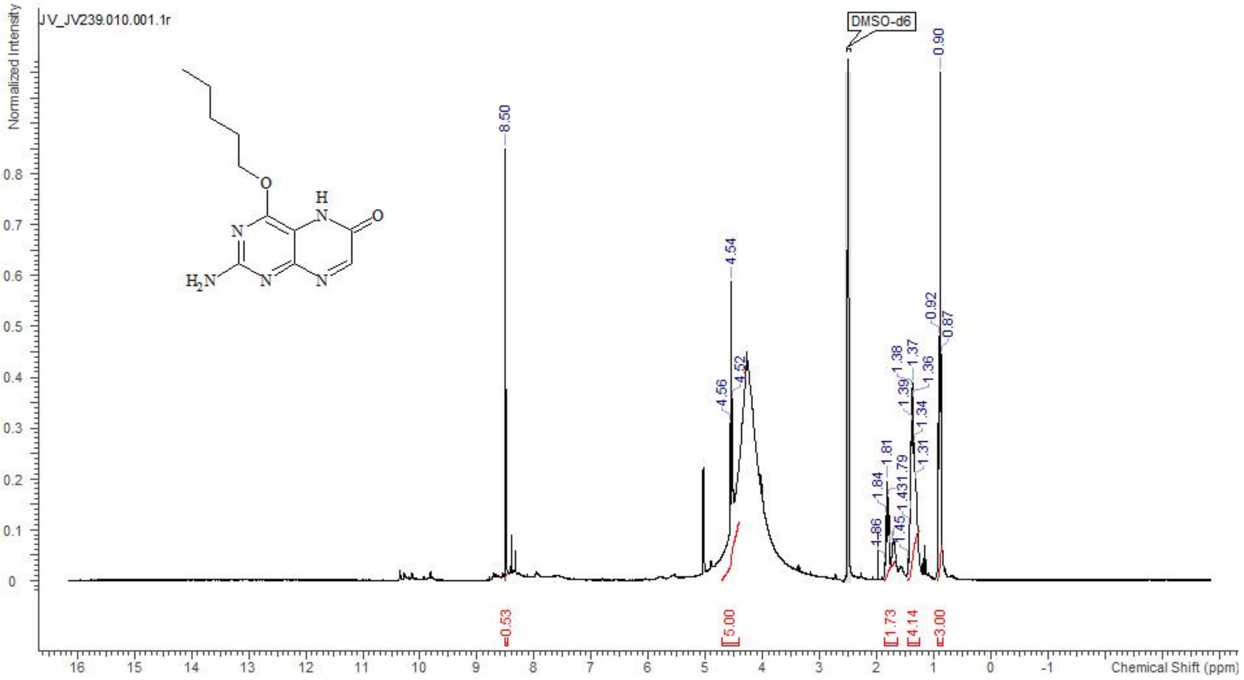

Figure 1: ^{1}H NMR (300 MHz, DMSO-d6) spectrum of compound **1**

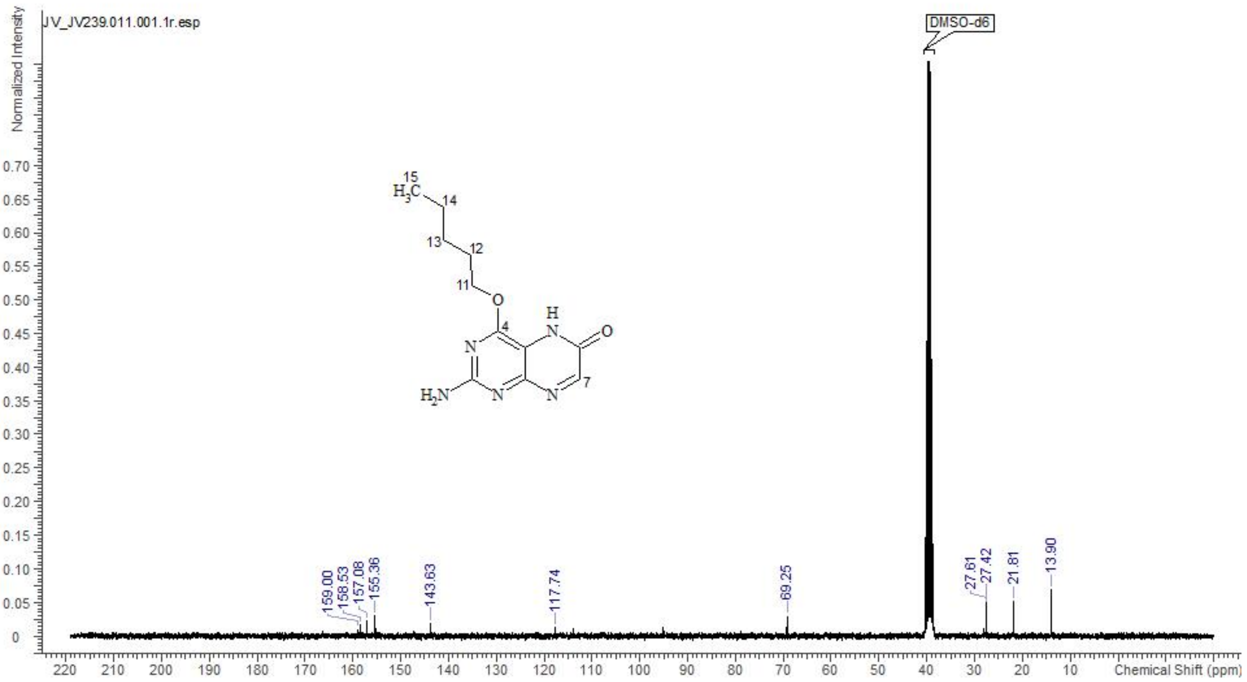

Figure 2: ^{13}C NMR (300 MHz, DMSO-d6) spectrum of compound **1**

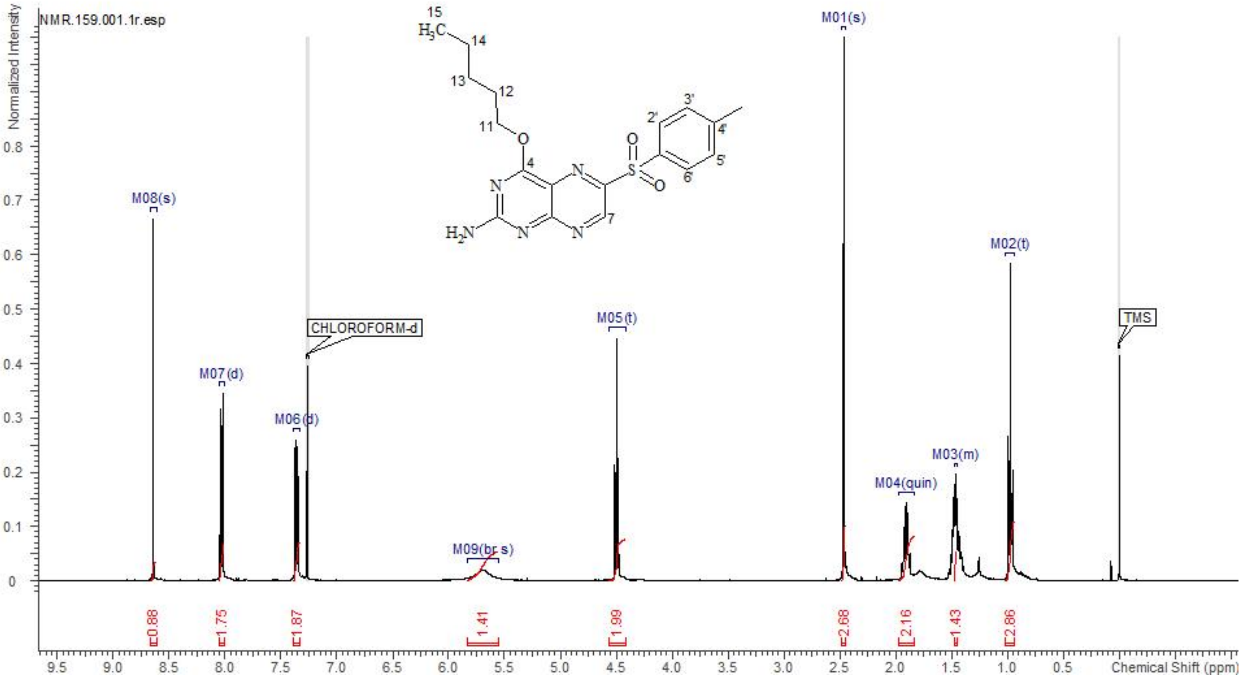

Figure 3: ^{1}H NMR (400 MHz, $CDCl_3$) spectrum of compound **2**

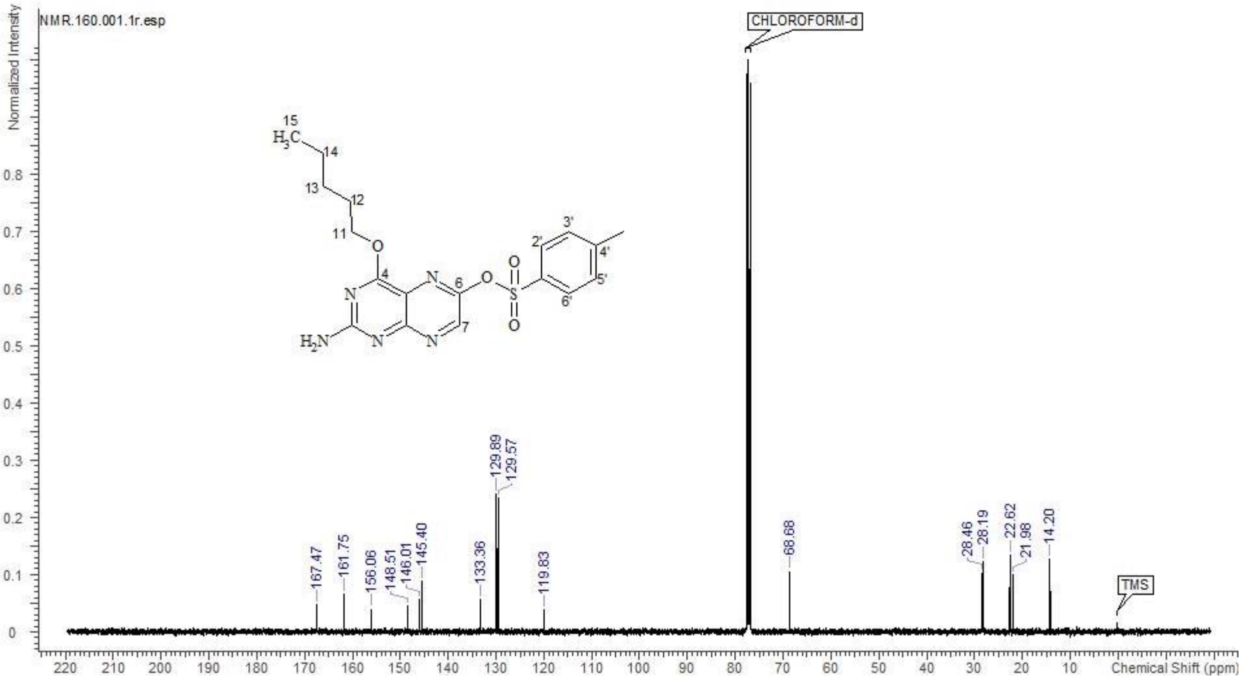

Figure 4: ^{13}C NMR (400 MHz, $CDCl_3$) spectrum of compound **2**

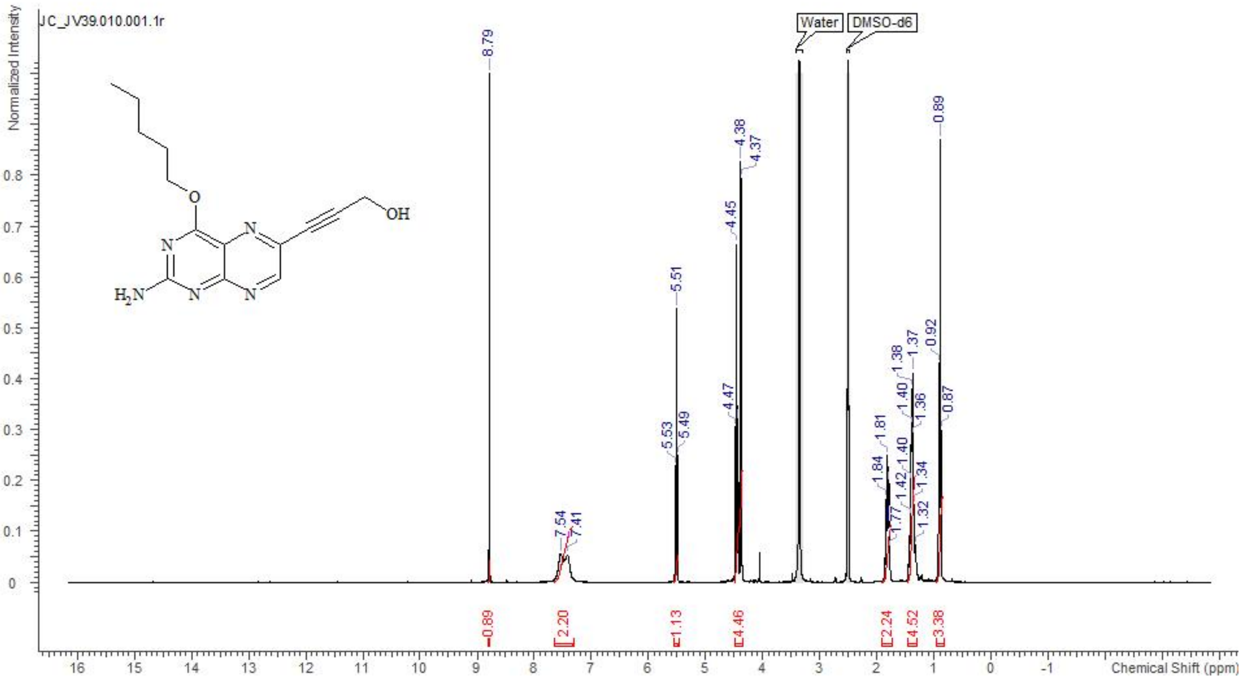

Figure 1: ^{1}H NMR (300 MHz, $CDCl_3$) spectrum of compound **3**

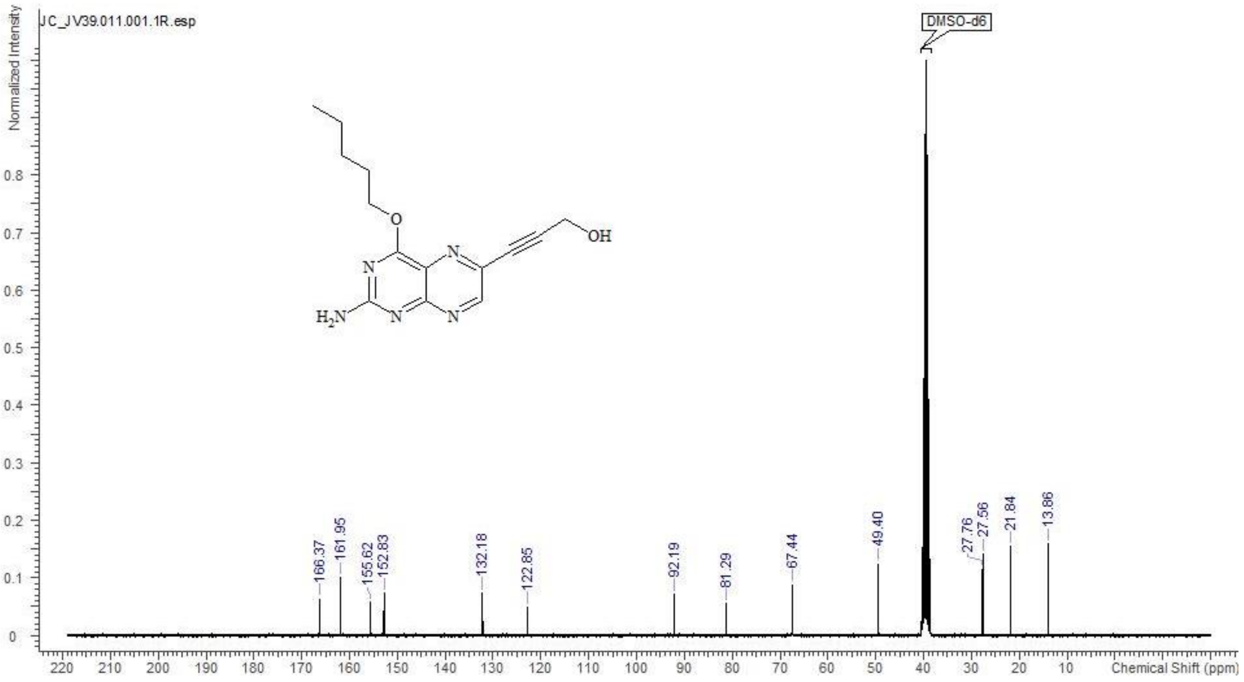

Figure 2: ^{13}C NMR (300 MHz, $CDCl_3$) spectrum of compound **3**

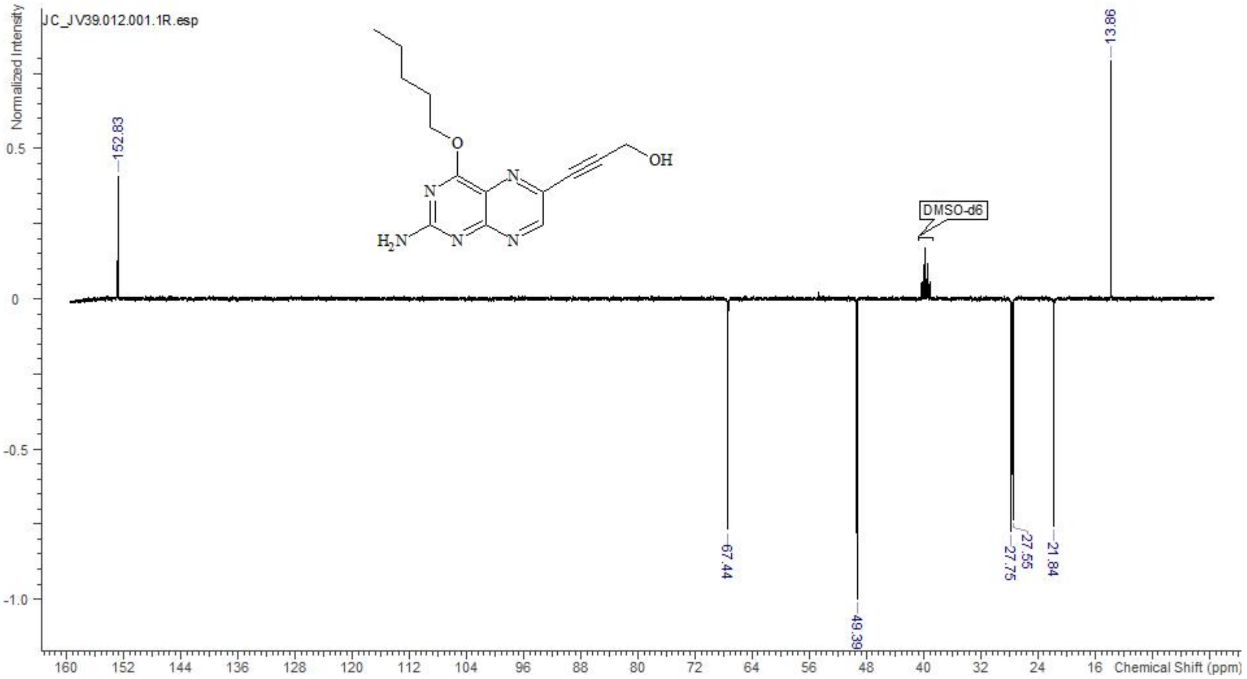

Figure 3: ^{13}C DEPT135 NMR (300 MHz, $CDCl_3$) spectrum of compound **3**

Compound 4

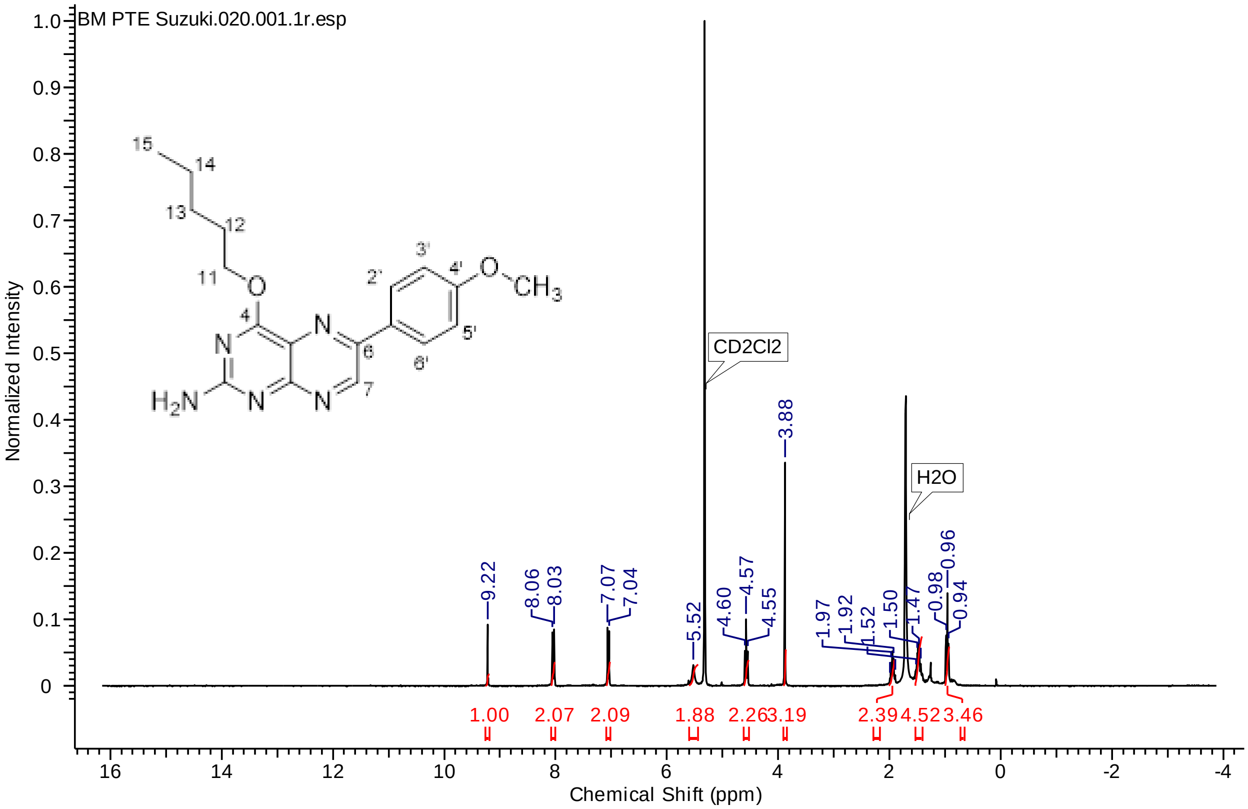

Figure 4: ^{1}H NMR (300 MHz, CD_2Cl_2) spectrum of compound ***4***

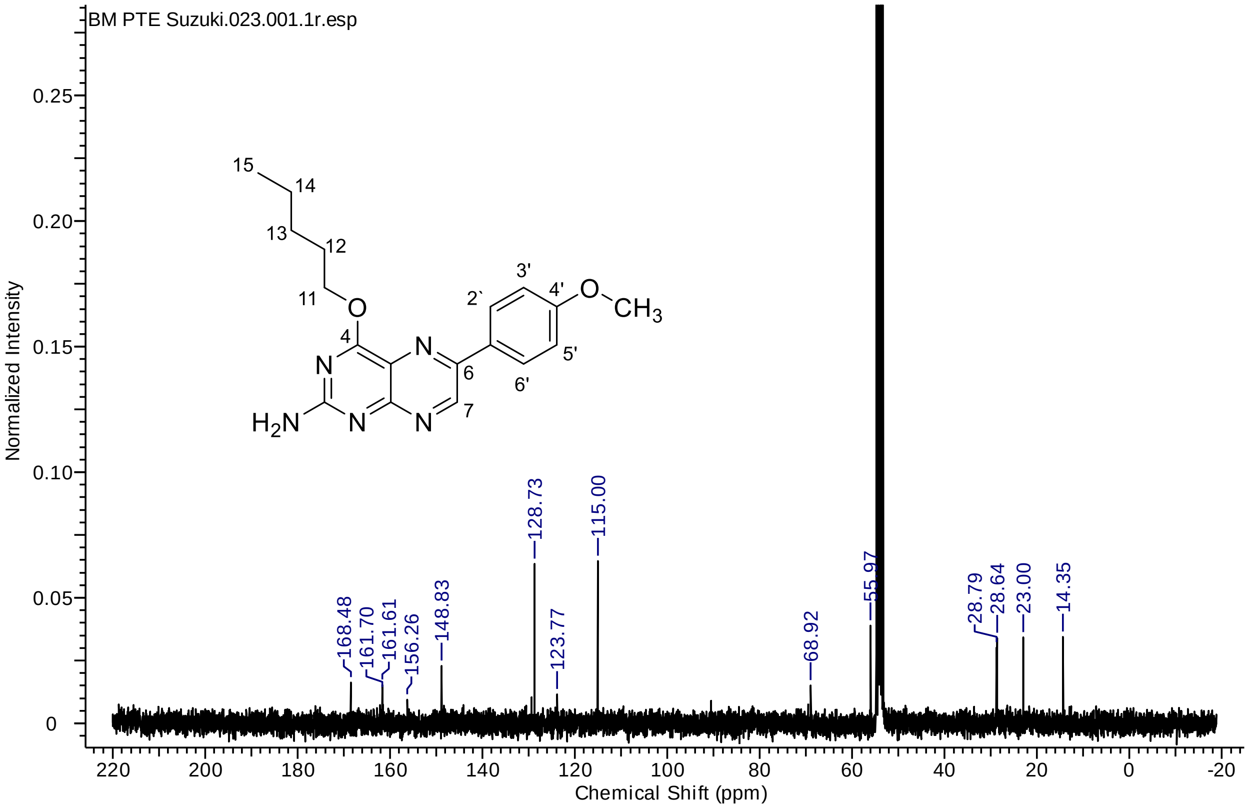

Figure 5: ^{13}C NMR (300 MHz, CD_2Cl_2) spectrum of compound **4**

Compound **5’**

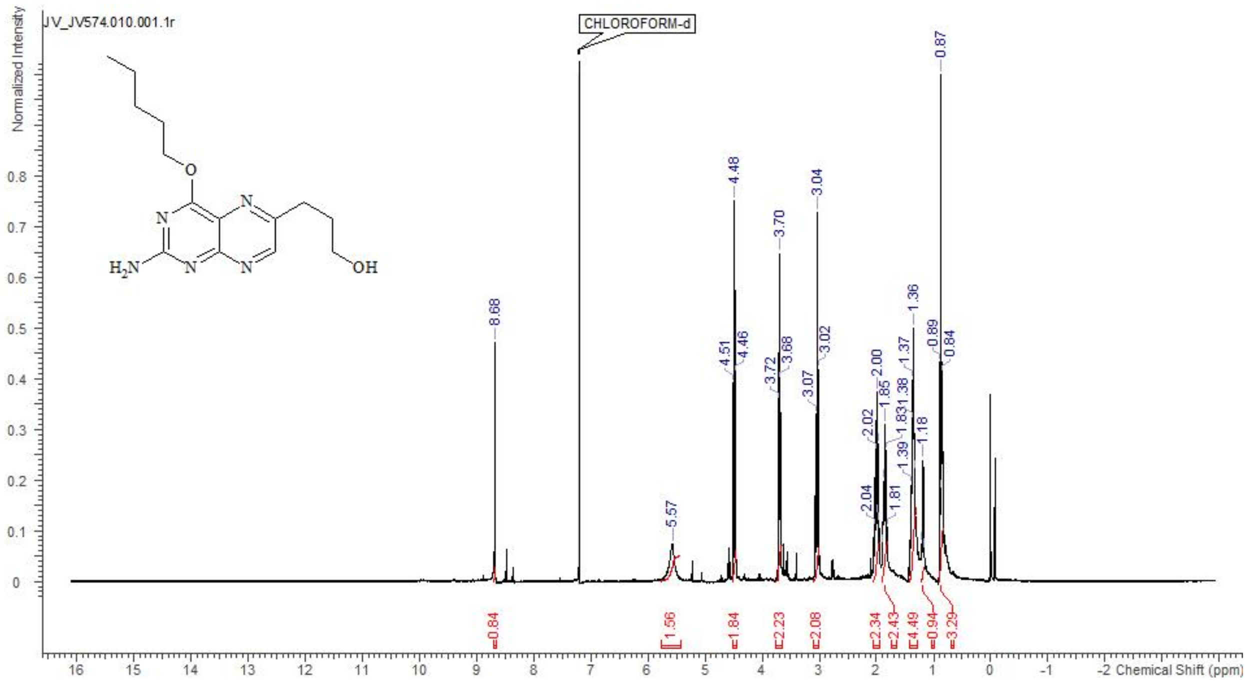

Figure 6: ^{1}H NMR (300 MHz, $CDCl_3$) spectrum of compound **5’**

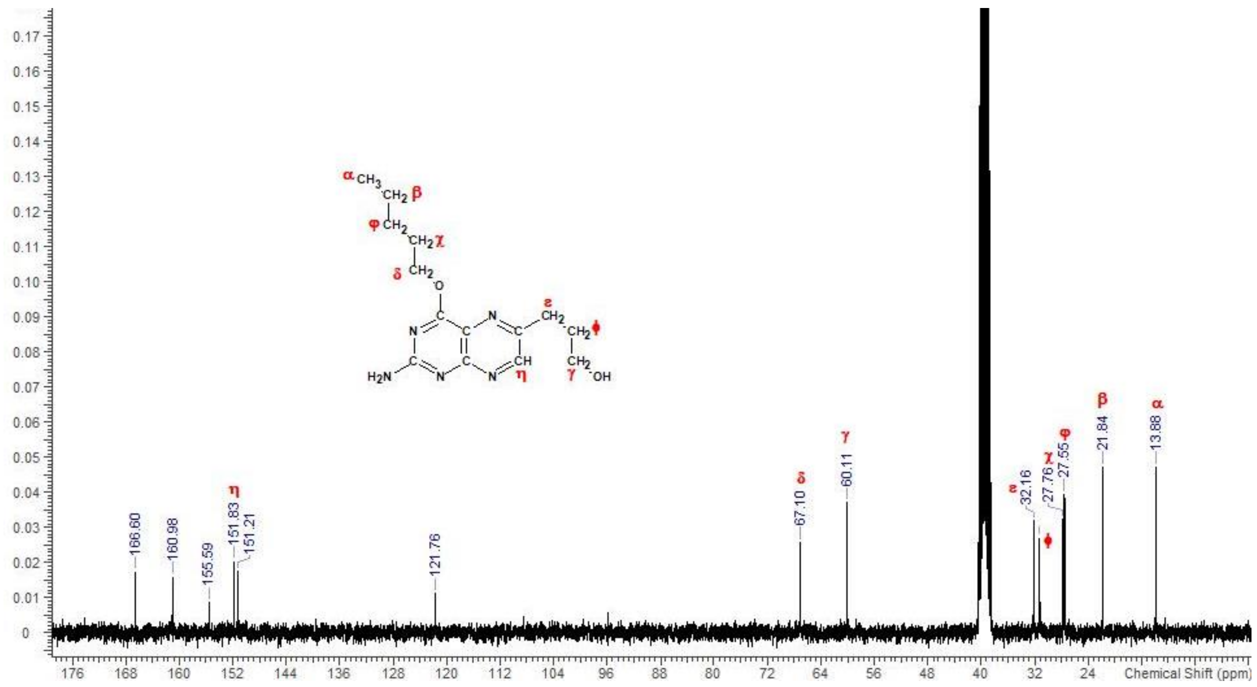

Figure 7: ^{13}C NMR (300 MHz, $CDCl_3$) spectrum of compound **5’**

Mass Spectra

Compound 1

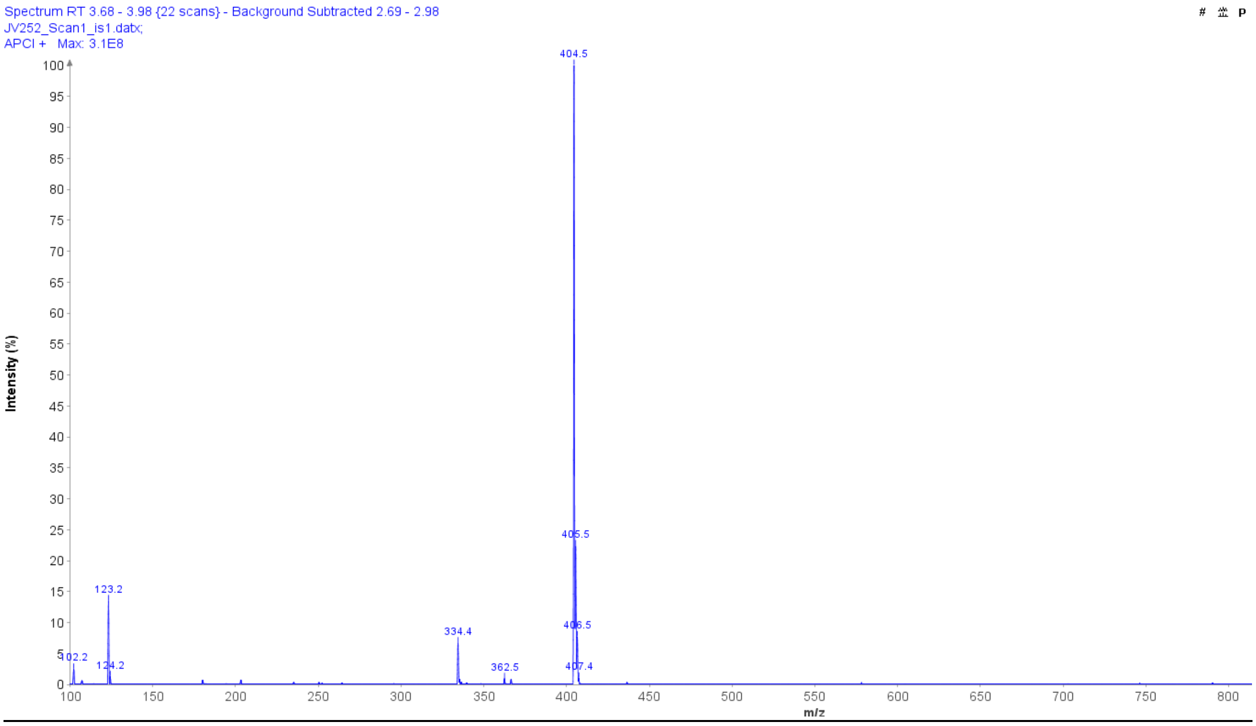

Compound 3

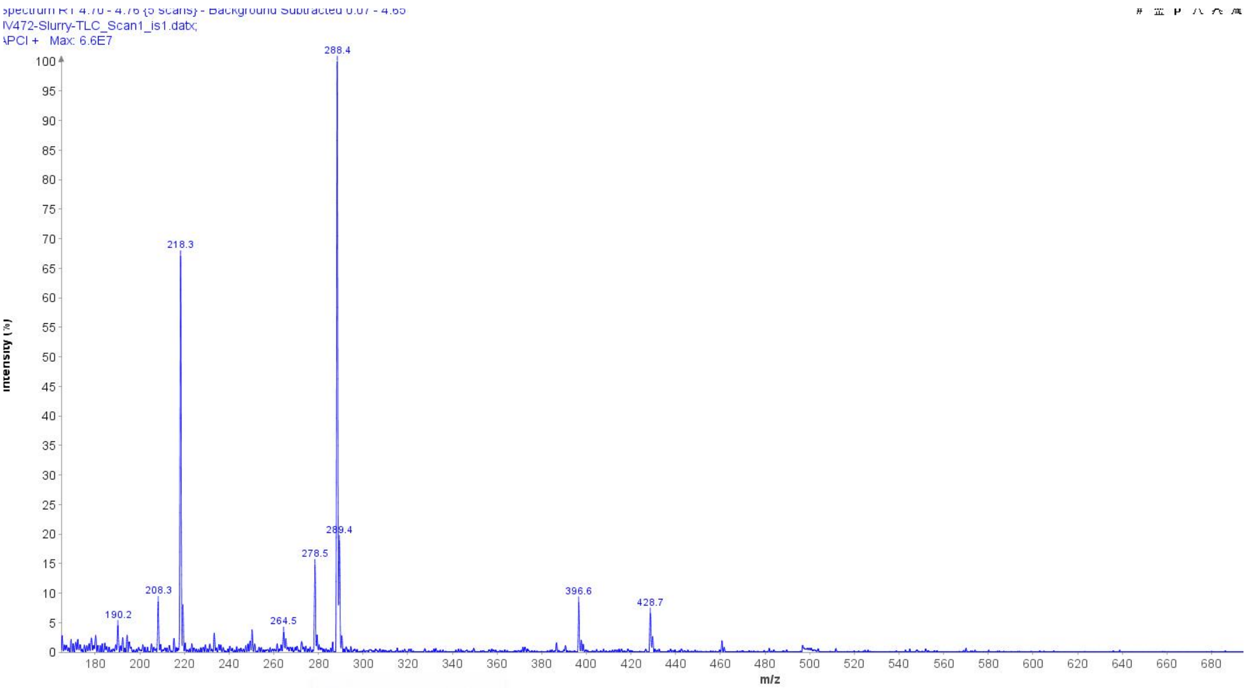

spectrum RT 4.70 - 4.76 {5 scans} - Background Subtracted 0.07 - 4.65
IV472-Slurry-TLC_Scan1_is1.datx;
APCI + Max: 6.6E7
Intensity (%)
m/z
288.4
218.3
289.4
278.5
208.3
190.2
264.5
396.6
428.7

Compound 4

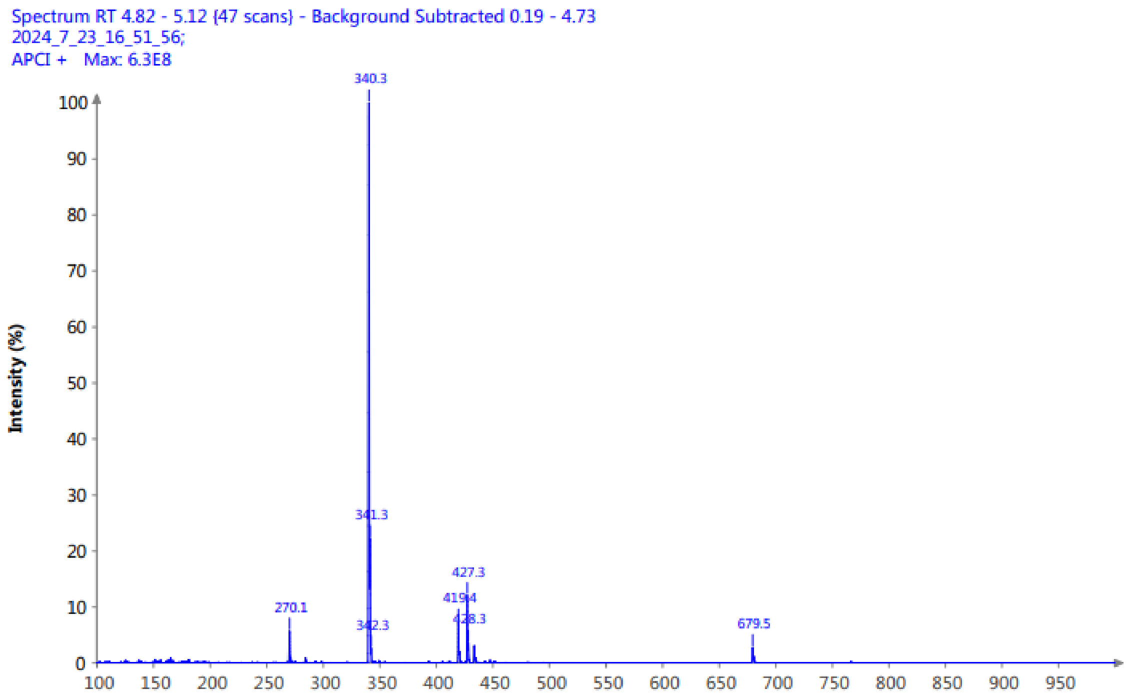
Spectrum RT 4.82 - 5.12 (47 scans) - Background Subtracted 0.19 - 4.73
2024_7_23_16_51_56;
APCI + Max: 6.3E8
Intensity (%)
340.3
341.3
342.3
270.1
427.3
419.4
428.3
679.5

Compound 5’

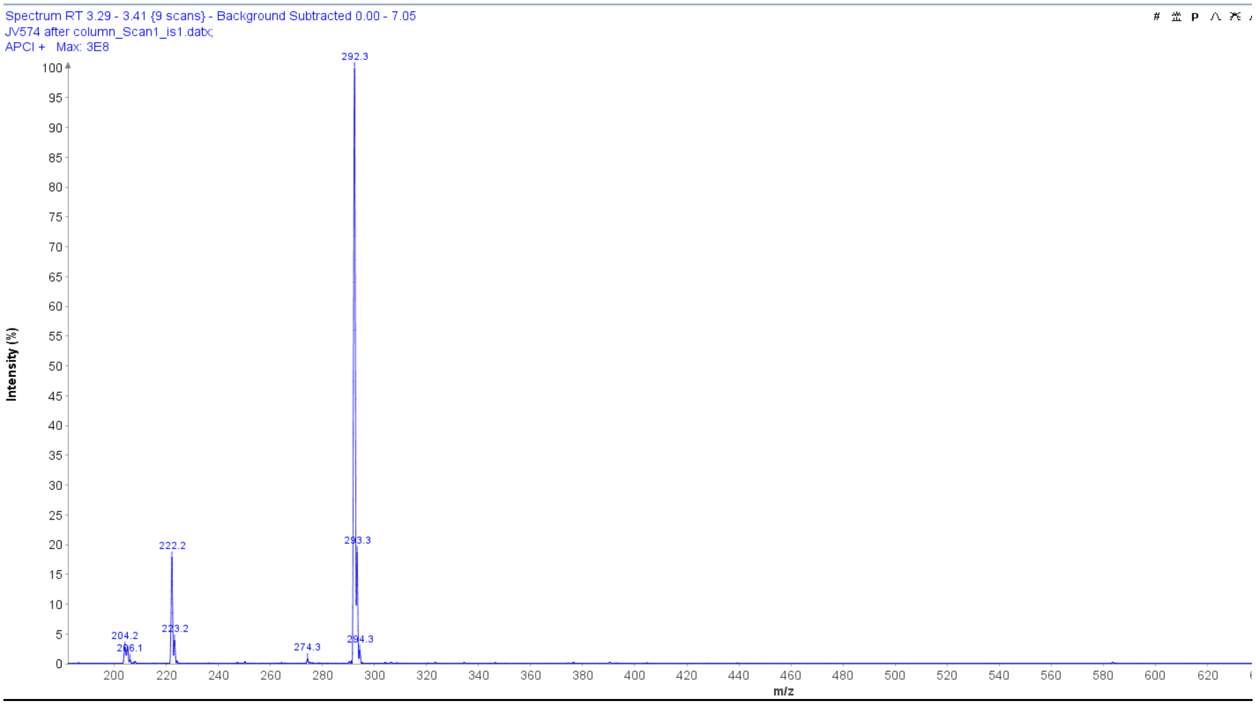
Spectrum RT 3.29 - 3.41 {9 scans} - Background Subtracted 0.00 - 7.05
JV574 after column_Scan1_is1.datx;
APCI + Max: 3E8
292.3
222.2
293.3
204.2
206.1
223.2
274.3
294.3
Intensity (%)
m/z

Compound 5

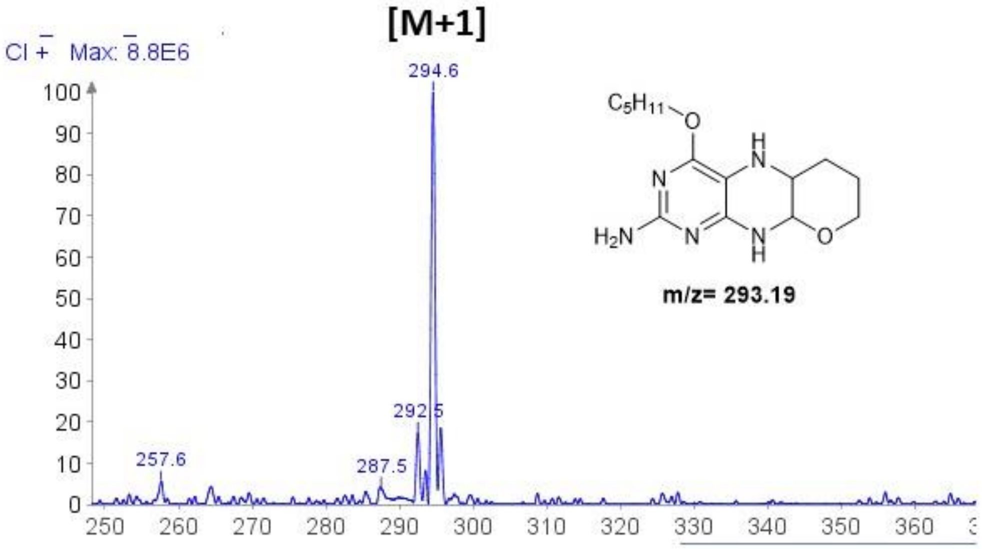

[M+1]
CI + Max: 8.8E6
294.6
292.5
287.5
257.6
C_5H_{11}
O
H
N
N
H_2N
N
N
H
O
m/z= 293.19

APCI-MS spectra of reduction reactions

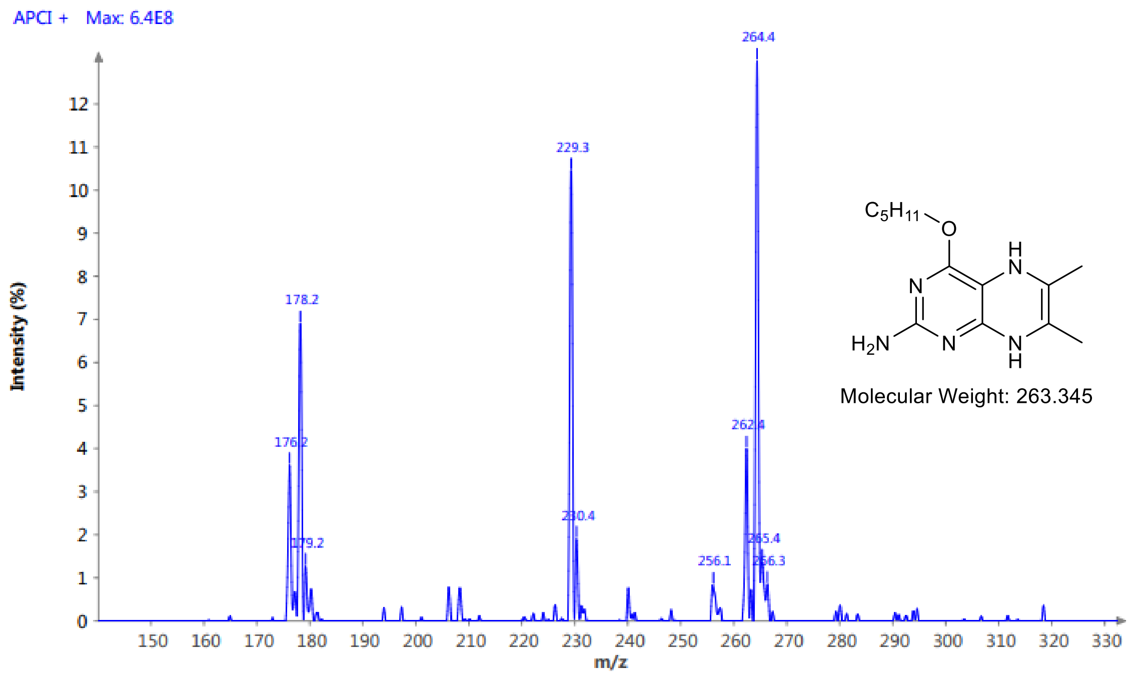

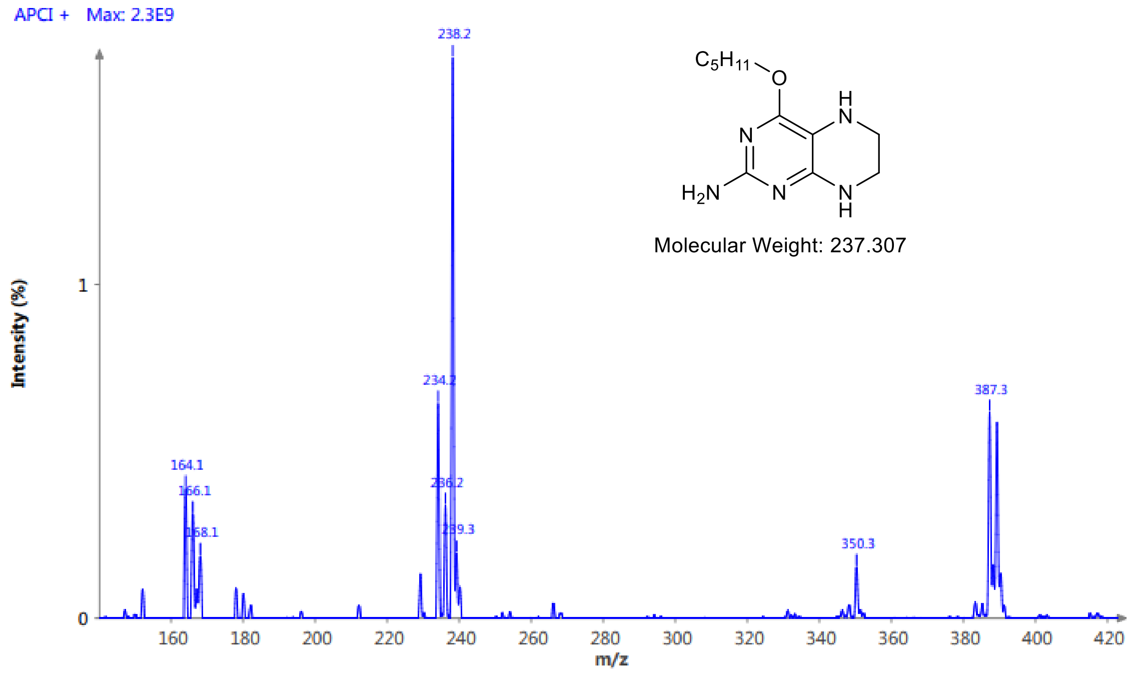

APCI +
Max: 2.3E9
238.2
234.2
236.2
239.3
164.1
166.1
168.1
350.3
387.3
Intensity (%)
m/z
C5H11
O
H
N
N
H2N
N
N
H
Molecular Weight: 237.307

Crystallographic data

Compound 2

**Identification code**	JV272B
**Empirical formula**	C18 H21 N5 O4 S
**Formula weight**	403.46
**Temperature**	170(2) K
**Wavelength**	0.71073 A
**Crystal system**	Triclinic,
**Space group**	P -1
**Unit cell dimensions**	a =9.2429(18) A alpha = 86.51(3) deg. b = 14.052(3) A beta =85.22(3) deg. c = 15.617(3) A gamma = 73.11(3) deg deg
**Volume**	1932.7(7) A^3
**Z, Calculated density**	4, 1.387 Mg/m^3
**Absorption coefficient**	0.203 mm^-1
**F(000)**	848
**Crystal size**	0.119 x 0.109 x 0.031 mm
**Theta range for data collection**	2.041 to 29.466 deg.
**Limiting indices**	-12<=h<=12, -18<=k<=19, -21<=l<=21
**Reflections collected / unique**	22364 / 10627 [R(int) = 0.0517]]
**Completeness to theta = 25.023**	99.8 %
**Absorption correction**	Numerical
**Max. and min. transmission**	0.9742 and 0.8575
**Refinement method**	Full-matrix least-squares on F^2
**Data / restraints / parameters**	10627 / 362 / 608
**Goodness-of-fit on F^2**	1.019
**Final R indices [I>2sigma(I)]**	R1 = 0.0470, wR2 = 0.1272
**R indices (all data)**	R1 = 0.0916, wR2 = 0.1509
**Extinction coefficient**	n/a
**Largest diff. peak and hole**	0.363 and -0.595 e.A^{-3}

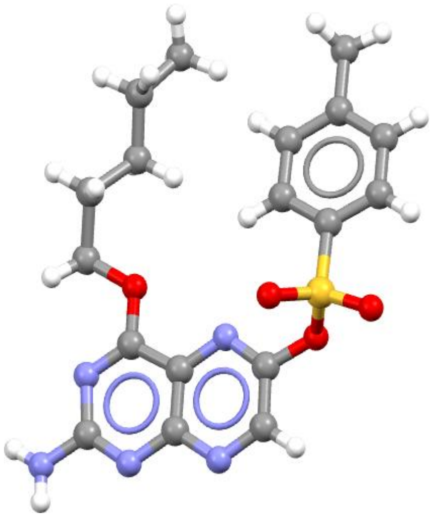

Compound 3

**Identification code**	JV39
**Empirical formula**	C14 H17 N5 O2
**Formula weight**	287.32
**Temperature**	170(2) K
**Wavelength**	0.71073 A
**Crystal system**	Triclinic,
**Space group**	P -1
**Unit cell dimensions**	a=4.9121(10) A alpha = 91.32(3) deg. b=11.649(2) A beta = 96.91(3) deg. c = 12.858(3) A gamma = 101.82(3) deg
**Volume**	714.1(3) A^3
**Z, Calculated density**	2, 1.336 Mg/m^3
**Absorption coefficient**	0.094 mm^-1
**F(000)**	304
**Crystal size**	0.501 x 0.123 x 0.041 mm
**Theta range for data collection**	3.196 to 26.368 deg.
**Limiting indices**	-12<=h<=12, -18<=k<=19, -21<=l<=21
**Reflections collected / unique**	5910 / 2890 [R(int) = 0.0503]
**Completeness to theta = [illegible]**	99.5 %
**Absorption correction**	Numerical
**Max. and min. transmission**	0.9774 and 0.6221
**Refinement method**	Full-matrix least-squares on F^2
**Data / restraints / parameters**	2890 / 96 / 232
**Goodness-of-fit on F^2**	0.955
**Final R indices [I>2sigma(I)]**	R1 = 0.0452, wR2 = 0.1076
**R indices (all data)**	R1 = 0.0931, wR2 = 0.1268
**Extinction coefficient**	n/a
**Largest diff. peak and hole**	0.222 and -0.221 $e.A^{-3}$

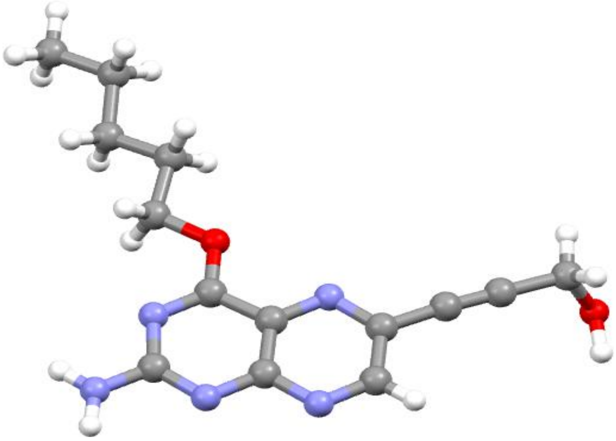

Compound 4

**Identification code**	SIV-BMSU
**Empirical formula**	C37 H42 Cl2 D2 N10 O4
**Formula weight**	765.73
**Temperature**	299(2) K
**Wavelength**	1.54184 A
**Crystal system**	Triclinic,
**Space group**	P -1
**Unit cell dimensions**	a= 8.3036(2) A alpha= 89.677(2) deg. b= 12.7600(3) A beta= 79.653(2) deg. c = 18.8608(4) A gamma = 79.304(2) deg.
**Volume**	1930.93(8) A^3
**Z, Calculated density**	2, 1.317 Mg/m^3
**Absorption coefficient**	1.945 mm^-1
**F(000)**	804
**Crystal size**	0.290 x 0.200 x 0.120 mm
**Theta range for data collection**	3.527 to 65.518 deg.
**Limiting indices**	-9<=h<=9, -14<=k<=15, -22<=l<=22
**Reflections collected / unique**	60235 / 6618 [R(int) = 0.1254]
**Completeness to theta = 25.023**	0.0 %
**Absorption correction**	Numerical
**Max. and min. transmission**	1.000 and 0.481
**Refinement method**	Full-matrix least-squares on F^2
**Data / restraints / parameters**	6618 / 409 / 606
**Goodness-of-fit on F^2**	1.058
**Final R indices [I>2sigma(I)]**	R1 = 0.0995, wR2 = 0.2342
**R indices (all data)**	R1 = 0.1223, wR2 = 0.2532
**Extinction coefficient**	0.0013(3)
**Largest diff. peak and hole**	0.288 and -0.215 $e.A^{-3}$

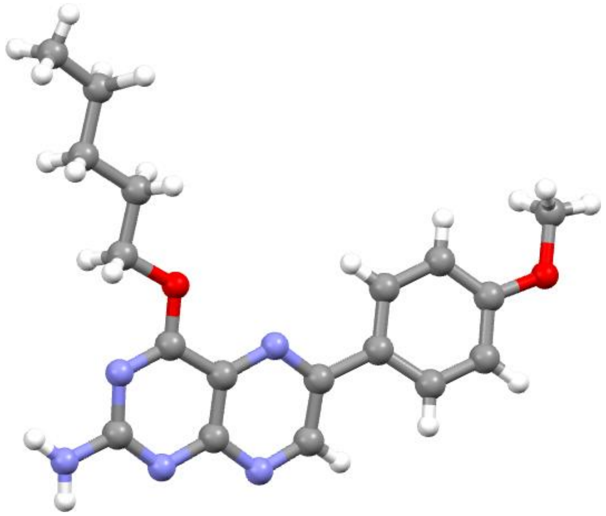

Supplement: Supplementary file 1 [file molecules-29-04587-s001.zip › Supplementary Information_24_09_13_rev.pdf]
